# LncRNA MM2P-induced, exosome-mediated transfer of Sox9 from monocyte-derived cells modulates primary chondrocytes

**DOI:** 10.1038/s41419-020-02945-5

**Published:** 2020-09-16

**Authors:** Jinyu Bai, Yingzi Zhang, Xin Zheng, Man Huang, Weinan Cheng, Huajian Shan, Xiang Gao, Mingchao Zhang, Lei Sheng, Jun Dai, Yekun Deng, Hong Zhang, Xiaozhong Zhou

**Affiliations:** 1 Department of Orthopedics, The Second Affiliated Hospital of Soochow University, Suzhou, Jiangsu 215004 China; 2 Department of Hematology, Suzhou Dushuhu Public Hospital (Dushuhu Public Hospital Affiliated to Soochow University, The First Affiliated Hospital of Soochow University, Dushuhu Branch, Suzhou, Jiangsu 215000 China; 3 Department of Obstetrics and Gynecology, The Second Affiliated Hospital of Soochow University, Suzhou, Jiangsu 215004 China

**Keywords:** Cell biology, Molecular biology

## Abstract

Monocyte-derived cells were shown to promote cartilage repair in osteoarthritis. The role of the long non-coding RNA (lncRNA) MM2P in this function of monocyte-derived cells remained unexplored. Treatment of RAW264.7 murine macrophages and mouse bone marrow-derived macrophages with IL-4 or IL-13 upregulated MM2P expression, upstream of STAT3 and STAT6 phosphorylation. Specifically, MM2P blocked SHP2-mediated dephosphorylation of STAT3 at Try705 and interacted with the RNA-binding protein FUS. In turn, p-STAT3 increased the Sox9 gene expression. These cells released Sox9 mRNA and protein-containing exosomes, as demonstrated by a transmission electron microscope, nanoparticle tracking analysis, and detection of typical surface markers. Their culture supernatant promoted the differentiation of mouse primary chondrocytes, i.e., upregulated the expression of Col1a2 and Acan genes and promoted the secretion of extracellular matrix components proteoglycan and type II collagen. These effects were mediated by Sox9 mRNA and protein delivered to chondrocytes by exosomes. Together, ex vivo treatment of monocyte-derived cells with IL-4 or IL-13 promoted chondrocyte differentiation and functions through exosome-mediated delivery of Sox9 mRNA and protein.

## Introduction

Osteoarthritis (OA) is the most common degenerative disorder of the joint, which is mainly attributed to an imbalance between repair and breakdown of the joint tissues or abnormal endochondral ossification due to mechanical loading^1,2^. Cartilage repair is suggested to be crucial to OA, however, the detailed mechanism of cartilage repair remains to be further clarified^3–5^.

Chondrocytes are known to be the resident cells responsible for maintaining a dynamic equilibrium between catabolism and anabolism in the extracellular matrix (ECM)^6^. Macrophages accumulate and polarize (M1 or M2) in the synovium and articular cavity during OA development^7^. The activated innate immune cells, especially macrophages, participate in OA progression^8^. Macrophages can be activated by certain biomaterials to release specific cytokines and possess immunomodulatory impacts on in vitro tissue recovery and osteogenic differentiation^9–11^. These findings indicate that macrophages M2 phenotype polarization may promote the articular homeostasis and regeneration in OA.

Long non-coding RNAs (lncRNAs) is a group of non-protein-coding transcripts containing over 200 nucleotides. Their roles in OA progression and cartilage repair have been emerging^12,13^. A recent study shows an abundant expression of lncRNA MM2P contributes to M2 polarization in macrophages^14^. This discovery reminds whether MM2P could promote cartilage repair through triggering M2 polarization.

Signal transducer and activator of transcription family (STATs) had important roles in M2 polarization^15,16^. STAT3 is a transcription factor belonging to the STATs family that is particularly studied in M2 polarization of macrophages^17,18^. In specific, STAT3 is activated through phosphorylation and undergoes dimerization and nuclear translocation to induce transactivation of target genes^19^. As repressors of STAT3 activation, protein tyrosine phosphatase (PTPs) are acknowledged^20^. Members of PTPs, such as SHP1 and SHP2, modulated the tyrosine dephosphorylation of STAT3 to repress STAT3 activity^21^. STAT3 has also been reported to activate SRY-box transcription factor 9 (SOX9) through binding to its proximal promoter to facilitate SOX9 transcription in chondrocytes^22^. SOX9 is a well-established regulator of cartilage repair and recognized to be a master regulator of several chondrogenic factors, including Col2a1^23^, Acan^24^, and Col1a1^25^. However, the relationship among MM2P, STAT3, and SOX9 is unclear.

Exosomes, a set of small membrane vesicles with 30–100 nm in size, can be released by most cells^26^. Exosomes are referred to as key intercellular communicators, since they transfer diverse types of molecules to recipient cells. These molecules include lncRNAs, microRNAs (miRNAs), messenger RNAs (mRNAs), and proteins^27^. Macrophages secret and transmit certain functional proteins and RNAs to affect various kinds of cells, such as fibroblast and epithelial cells^28,29^. Previous research demonstrates that osteoarthritic chondrocytes secret exosome-like vesicles to facilitate the production of IL-1β in macrophages and aggravate OA progression^30^. However, whether macrophage-derived exosomes regulate cartilage repair is unknown. Moreover, whether MM2P regulates the effect of macrophage-derived exosomes on cartilage repair has never been investigated. In this study, we clarify whether MM2P promotes cartilage repair through M2 polarization.

## Materials and methods

### Cell culture and reagent

RAW264.7 cells (ATCC, Rockville, Maryland) were maintained in high-glucose DMEM (HDMEM, Hyclone, Logan, UT) adding 10% fetal bovine serum (FBS; HyClone) and antibiotics (penicillin–streptomycin; HyClone). The extracted newborn mouse articular chondrocytes were cultivated in HDMEM and used for study after one to three passages. All cell cultures were performed at 37 °C in a 5% CO_2_ atmosphere. For treating cells, the recombinant murine IL-4 and IL-13 were acquired from PeproTech (Rocky Hill, NJ). 20 μg/mL of ActynomicinD (ActD) from Sigma-Aldrich (St. Louis, MO) was applied to detect mRNA stability. 10 mg/mL of RNase A and 0.5% Triton-X 100 were both acquired from ThermoFisher Scientific (Waltham, MA).

### Isolation and differentiation of bone marrow-derived macrophages (BMDMs)

BMDMs were obtained obeying former descriptions^31^. C57BL/6 mice (6-week-old) were killed via cervical dislocation and then sterilized using 75% ethanol. After incising the skin at the root of the hind legs, muscle tissues were removed using scissors from the bone. Later, the bones cut from both ends were kept on ice and were rinsed with a macrophage complete medium (Gibico, Grand Island, NY, USA) using a 30-gauge needle in a sterile environment. The harvested cells were washed and then pipetted up and down in a culture dish to obtain a uniform suspension.

The bone marrow cells were allowed to differentiate into macrophages for 7 days in a culture medium at 37 °C with 5% CO_2_. The medium was changed every 48 h. The protocols for experiments on animals were approved by the ethics committee of the Second Affiliated Hospital of Soochow University. The percentage of markers of BMDMs was measured for further experiments. BMDMs were incubated in a blocking solution containing PBS, 0.1% Triton-X, 0.1% Tween-20, 2% bovine serum albumin, and 4% goat serum to prevent the non-specific binding for 1 h at room temperature. After removed the blocking solution, BMDMs were incubated with primary antibodies (anti-F4/80, anti-CD86, and anti-CD206, Abcam) at 1:200 dilutions for 16 h at 4 °C. BMDMs were incubated with the secondary antibodies (Abcam) for 1 h at room temperature followed by rinsing with PBS. Finally, BMDMs were subjected to the analysis with the BD FACS Calibur cytometer (Becton-Dickinson, Sparks, MD). The percentage of F4/80, CD86, or CD206-positive cells was calculated.

### Real-time quantitative PCR (RT-qPCR)

Total RNA samples were severally isolated by Trizol kit (GIBCO BRL, Gaithersburg, MD) and quantitated via spectrophotometer (Beckman, Fullerton, CA). The BioRad IQ5 thermocycler (BioRad Laboratories, Inc., Hercules, CA) was used to assess SYBR Green fluorescent dye (Invitrogen, Carlsbad, CA, USA) in double-strand DNA for qPCR. Threshold cycles (Ct) of detected RNA were standardized to housekeeping gene GAPDH, ACTB, or 18S rRNA for quantification.

### Flow cytometry

Cells were reaped with the scraper, blocked in 3% BSA for 45 min, and then cultured with PE-conjugated anti-mouse CD86 (1:200) or PE-conjugated anti-mouse CD206 (at dilution of 1:200) gated with FITC-conjugated anti-mouse F4/80 (1:200) in line with the guidelines. About 1 × 10^4^ cells in each sample were subjected to analysis of BD FACS Calibur cytometer (Becton-Dickinson, Sparks, MD).

### Transfection

Cells at about 70–80% confluence were plated in six-well culture plates and transfected with indicated plasmids for 48 h by applying Lipofectamine2000 reagent (Invitrogen). The specific short hairpin RNAs (shRNAs) for MM2P (sh#1/2) or FUS (sh-FUS), relative negative control (NC; sh-NC), the pcDNA3.1-STAT3, pcDNA3.1-SOX9, and empty vectors were all procured from RiboBio (Guangzhou, China).

### Western blot

Total proteins were extracted in RIPA lysis buffer (Beyotime, Shanghai, China) with the protease inhibitor cocktail (Roche, Pleasanton, CA, USA). Protein concentration was determined with the BCA Protein Assay Kit (Pierce, Rockford, IL, USA). Samples were loaded and separated by 10% SDS-PAGE gels, followed by transfer to polyvinylidene difluoride (PVDF) membranes (Millipore, Bedford, MA, USA). The membranes were blocked in 5% skimmed milk and then incubated with indicated primary antibodies and secondary antibody (Abcam, Cambridge, UK). At length, the signals in membranes were observed with the chemiluminescence (ECL) system (BioRad lab, Hercules, CA, USA).

### Immunofluorescence (IF)

Cells were first plated on culture slides for 14 h. Then cells were fixed with 4% (vol/vol) paraformaldehyde–PBS, permeabilized with 0.5% Triton-X 100–PBS, and blocked with 5% (wt/vol) BSA–PBS for 1 h. The slides were incubated with the specific primary antibodies for p-STAT3 and STAT3, followed by rinsing with PBS and stained with secondary antibodies. Fluorescent imaging was performed with a confocal laser-scanning microscope (Leica Microsystems, Wetzlar, Germany).

### Measurement of cytokines

The supernatant was collected from chondrocytes, glycosaminoglycan (GAG) production, and type II collagen (Col II) secretion were separately monitored by colorimetric assay kit (Millipore) and enzyme-linked immunosorbent assay kit (ELISA; BD Biosciences, San Jose, CA) following their relative manual.

### Isolation of exosomes and morphology size analysis

The RAW264.7 cells at 70% confluence were reaped and rinsed in PBS, then incubated for 48 h with 10% exosome-depleted FBS. The exosomes derived from RAW264.7 cells were named as RAW264.7 Exo. The morphology of exosomes was detected by Transmission electron microscopy (TEM) from Hitachi (Tokyo, Japan) and imaged by a digital camera (Olympus, Tokyo, Japan). The size and number of the exosomes were analyzed employing Nanoparticle Tracking Analysis (NTA) with Tunable Resistive Pulse Sensing (Christchurch, New Zealand).

### Establishment of animal model

As previously described, male C57BL/6 mice (2-months old) obtained from the Institute of Laboratory Animals Science (Beijing, China) were subjected to destabilization of the medial meniscus (DMM) surgery to establish the OA model^32^. The animal study followed the principle of randomization. Briefly, the mice were prepared for the surgery through intraperitoneal anesthesia with chloral hydrate (400 mg/kg). The right knee joint was exposed to the medial capsular incision. Meanwhile, a sham operation was performed as a control group. To assess the role of exosome-derived lncRNA MM2P, the OA mice model were randomly divided into four groups (PBS, Exo, Exo/sh-NC, and Exo/sh-MM2P). One week after DMM surgery, mice were treated through tail-vein injection. Three weeks later, the mice were killed by cervical dislocation and knee joints were collected for H&E staining or Saffron O staining as previously elucidated^6,33^.

### Chromatin immunoprecipitation (ChIP) analysis

ChIP assay was performed in light of the protocol of EZ ChIP™ Chromatin Immunoprecipitation Kit for cell line (Millipore). The treated cells were fixed in formaldehyde for crosslink and sonicated for shearing DNA, followed by incubation with indicated antibodies for 6 h. Normal mouse IgG acted as the NC. The precipitates from beads were analyzed by RT-qPCR.

### Dual-luciferase reporter assays

The amplified SOX9 promoter by PCR was inserted into the firefly plasmid in the pGL3-Basic vector (Promega, Madison, WI). Cells in 96-well plates were co-transfected with a pGL3-SOX9 promoter, pRL-TK-Renilla (Promega), and indicated transfection plasmids for 48 h. The relative luciferase activity was determined by the ratio of firefly to Renilla activity using the Dual-Luciferase Reporter Assay System (Promega).

### Exosome labeling

Exosomes were labeled using PKH67 Green Fluorescent Cell Linker Kit (Sigma-Aldrich) as per the instruction. The exosome pellets were cultured with 2 μL PKH67 for 6 h, then ultracentrifuged and rinsed. At length, labeled exosomes were analyzed by a laser-scanning confocal microscope (Leica Microsystems, Wetzlar, Germany).

### Subcellular fraction assay

Cells were re-suspended in fraction buffer for 10 min and homogenized to obtain the cytoplasm fraction (supernatant) after centrifugation. The remaining pellets were treated with nuclear isolation buffer for 20 min on ice for removing the residual cytoplasmic faction. Following RNA extraction from nuclear and cytoplasmic fractions, the cellular location of MM2P was determined by RT-qPCR, with the GAPDH or U6 as cytoplasmic and nuclear references.

### RNA pulldown assay

RNA pulldown assay was implemented in light of the manual of Pierce Magnetic RNA-Protein Pull-Down Kit (ThermoFisher Scientific). Cell lysates were collected for incubation with the indicated biotin-labeled RNA probes, in the presence of beads. After 1 h, the pulldown complex was subjected to RT-qPCR or western blot analysis.

### Fluorescence in situ hybridization (FISH)

MM2P FISH probes constructed at RiboBio (Guangzhou, China) were purchased for FISH assay. Cells fixed in 4% formaldehyde and washed with 0.5% Triton-X 100 in PBS. Then, the air-dried cells were hybridized with MM2P FISH probes. After washing and dehydrating, cell nuclei were stained with DAPI (Beyotime), followed by the application of a fluorescence microscope (Olympus).

### RNA immunoprecipitation (RIP) analysis

In strict accordance with the instruction of EZ-Magna RIP kit (Millipore), cells in the RIP lysis buffer were treated with beads conjugated to specific antibodies and anti-IgG as NC and finally assayed by RT-qPCR.

### Co-immunoprecipitation (Co-IP)

Cell lysates were acquired from the treated cells in IP lysis buffer for incubation with a specific antibody to SHP1 or SHP2 and anti-IgG (negative control) all night at 4 °C. Following washing thrice in IP lysis buffer, samples were boiled in SDS loading buffer and eluted for western blot.

### Statistical analyses

In this study, triplicates were required for all assays. Data analyses were conducted by *t*-test or one-way analysis of variance applying PRISM 6 (GraphPad, San Diego, CA), normalized to the threshold of *p*-value < 0.05. Continuous variables were shown as mean ± standard deviation (SD).

## Results

### MM2P facilitated M2 polarization

**Fig. 1:**
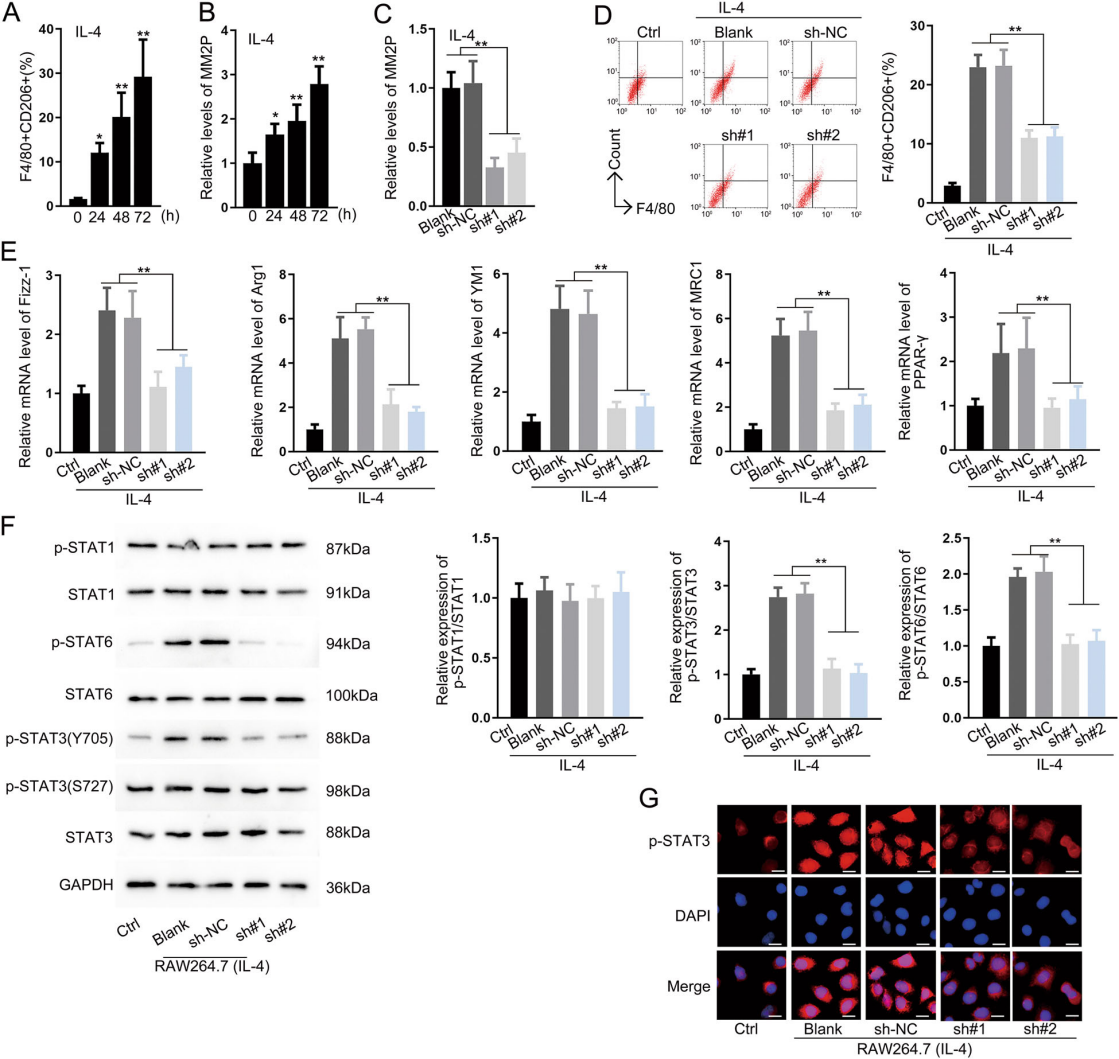
MM2P facilitated M2 polarization. **a** Flow cytometry analysis for the ratio of F4/80^+^CD206^+^ in RAW264.7 cells after treatment with IL-4. **b** Level of MM2P following the induction of M2 polarization at the indicated time. **c** The effect of sh-MM2P#1/2 transfection on the expression of MM2P in IL-4-induced M2 macrophages was measured by RT-qPCR. **d** Pictures of flow cytometry of F4/80^+^CD206^+^ cells and quantification of F4/80^+^CD206^+^ cell ratio under MM2P depletion in IL-4-induced cells. **e** RT-qPCR data for the levels of M2-related genes (Fizz-1, Arg1, YM1, MRC1, PPAR-γ) under the treatment of IL-4 with MM2P depletion in RAW264.7 cells. **f** Western blot for the total and phosphorylated STAT1, STAT6, and STAT3 in RAW264.7 cells treated with IL-4. **g** IF staining for the fluorescence intensity of p-STAT3 under IL-14 stimulation, as well as depletion of MM2P. Scale bar = 20 μm. **P < 0.01. Error bars are said to express as mean ± SD of three independent experiments in triplicates.

To confirm the function of MM2P in M2 polarization of RAW264.7 cells, 10 ng/mL IL-13 or IL-4 were applied to induce the M2 phenotype and the ratio of F4/80^+^CD206^+^ RAW264.7 cells increased gradually at 24, 48, and 72 h (Fig. 1a and Supplementary Fig. S1A). MM2P was upregulated following the induction of M2 polarization (Fig. 1b and Supplementary Figs. S1B and S2A). Transfection of sh-MM2P#1/2 downregulated MM2P (Fig. 1c and Supplementary Figs. S1C and S2B), and the ratio of F4/80^+^CD206^+^ was decreased (Fig. 1d and Supplementary Fig. S1D). In addition, M2-related genes, including *Fizz-1, Arg1, YM1, MRC1*, and *PPAR-γ* were downregulated under the silence of MM2P (Fig. 1e and Supplementary Figs. S1E and S2C). The expression of p-STAT3 (Y705) rather than p-STAT3 (S727) was increased under the treatment of IL-4 and IL-13. Nevertheless, there was no significant change in STAT1 and STAT6 (Fig. 1f and Supplementary Fig. S1F). As showed in Fig. 1g and Supplementary Fig. S1G, increased intensity of p-STAT3 was counteracted by the depletion of MM2P (Fig. 1g and Supplementary Fig. S1G).

**Fig. 2:**
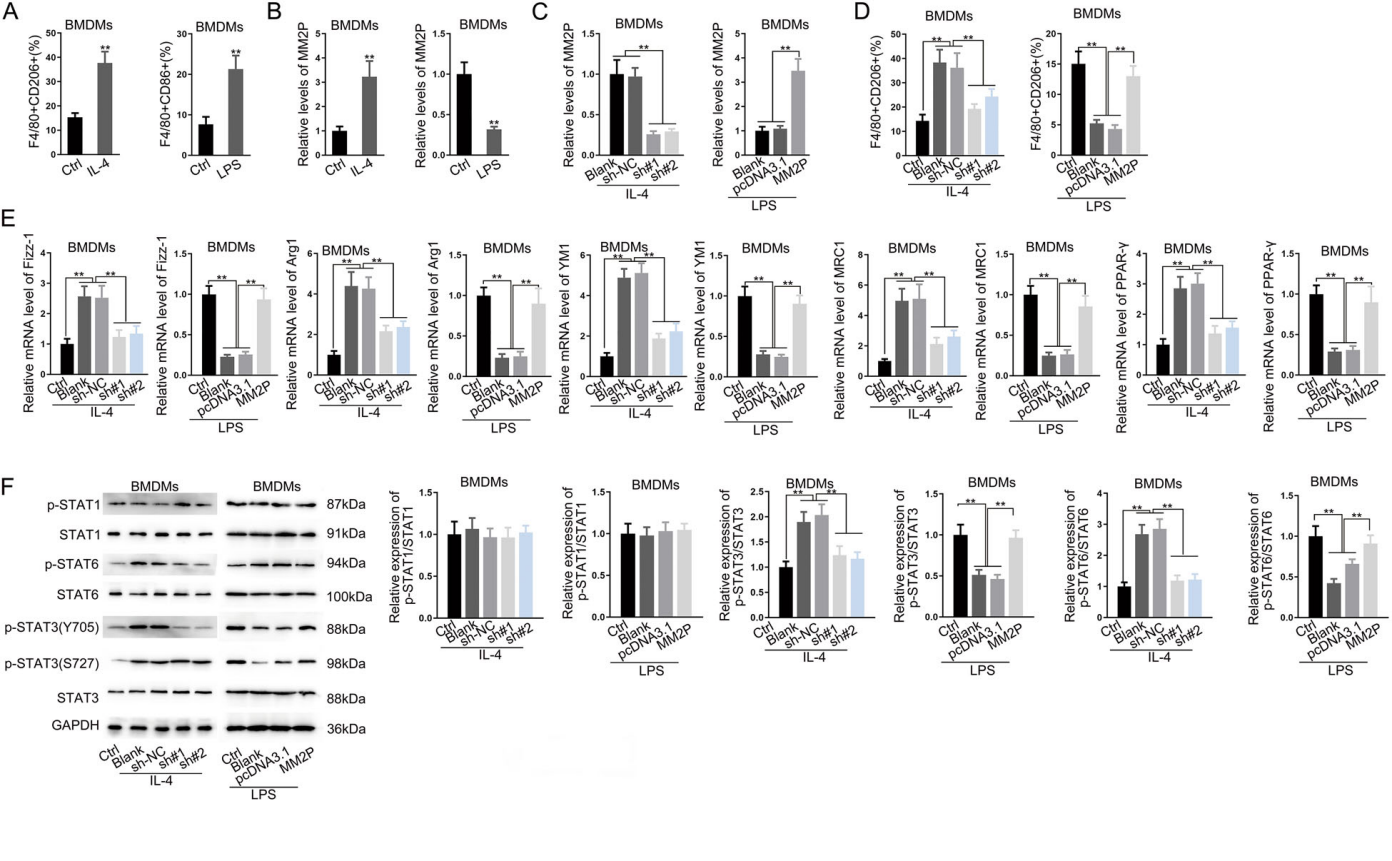
MM2P promoted switch from M1 to M2 phenotype to facilitate cartilage repair. BMDMs were treated with IL-4 or LPS to induce M2 or M1 polarization. **a** Quantification of F4/80^+^CD206^+^ ratio in IL-4-treated BMDMs, and F4/80^+^CD86^+^ ratio in LPS-treated BMDMs. **b** RT-qPCR of MM2P levels in BMDMs of each group. **c** The IL-4-treated BMDMs were transfected with sh-NC or sh-MM2P#1/2, whereas the LPS-treated BMDMs were transfected with pcDNA3.1 or pcDNA3.1/MM2P. RT-qPCR of MM2P levels in BMDMs of each group. **d** Quantification of F4/80^+^CD206^+^ ratio in IL-4-treated BMDMs, and F4/80^+^CD86^+^ ratio in LPS-treated BMDMs. **e** RT-qPCR of MM2P levels in BMDMs of each group. **f** Western blots of p-STAT1, STAT1, p-STAT6, STAT6, p-STAT3, STAT3, and quantification of p-STAT1/STAT1, p-STAT6/STAT6, and p-STAT3/STAT3 in BMDMs treated with IL-4 and LPS. **P < 0.01. The error bar expressed as mean ± SD of three independent experiments in triplicates.

**Fig. 3:**
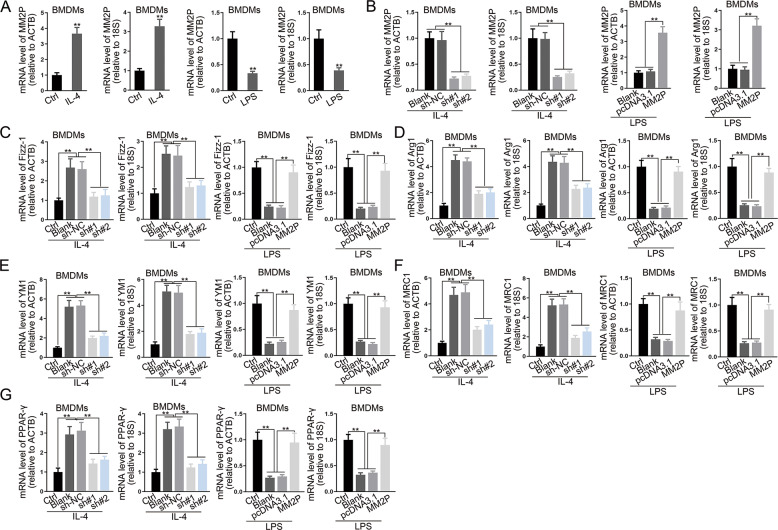
RT-qPCR analysis of lncRNA MM2P or the macrophage-related genes in LPS/IL-4-induced BMDMs transfected with MM2P-specific shRNA or MM2P expression vector using ACTB and 18S as housekeeping genes. **P < 0.01. The error bar expressed as mean ± SD of three independent experiments in triplicates.

IL-4 induced M2 phenotype, whereas LPS induced the M1 phenotype in BMDMs (Fig. 2a). IL-4 increased, whereas LPS reduced the MM2P level (Figs. 2b and 3a). The knockdown of MM2P reduced the ratio of F4/80^+^CD206^+^, whereas overexpression of MM2P increased (Fig. 2d). M2-related genes were downregulated by MM2P knockdown in IL-4-treated BMDMs and increased by MM2P overexpression in LPS-treated BMDMs (Figs. 2e and 3c–g). Moreover, we found that the level of p-STAT3 (Y705) and p-STAT6 showed similar consequences (Fig. 2f). These results indicated that MM2P facilitated M2 polarization through STAT6 and STAT3 signaling.

### Exosomes from M2 phenotype macrophages promoted chondrocyte-specific genes and proteins

**Fig. 4:**
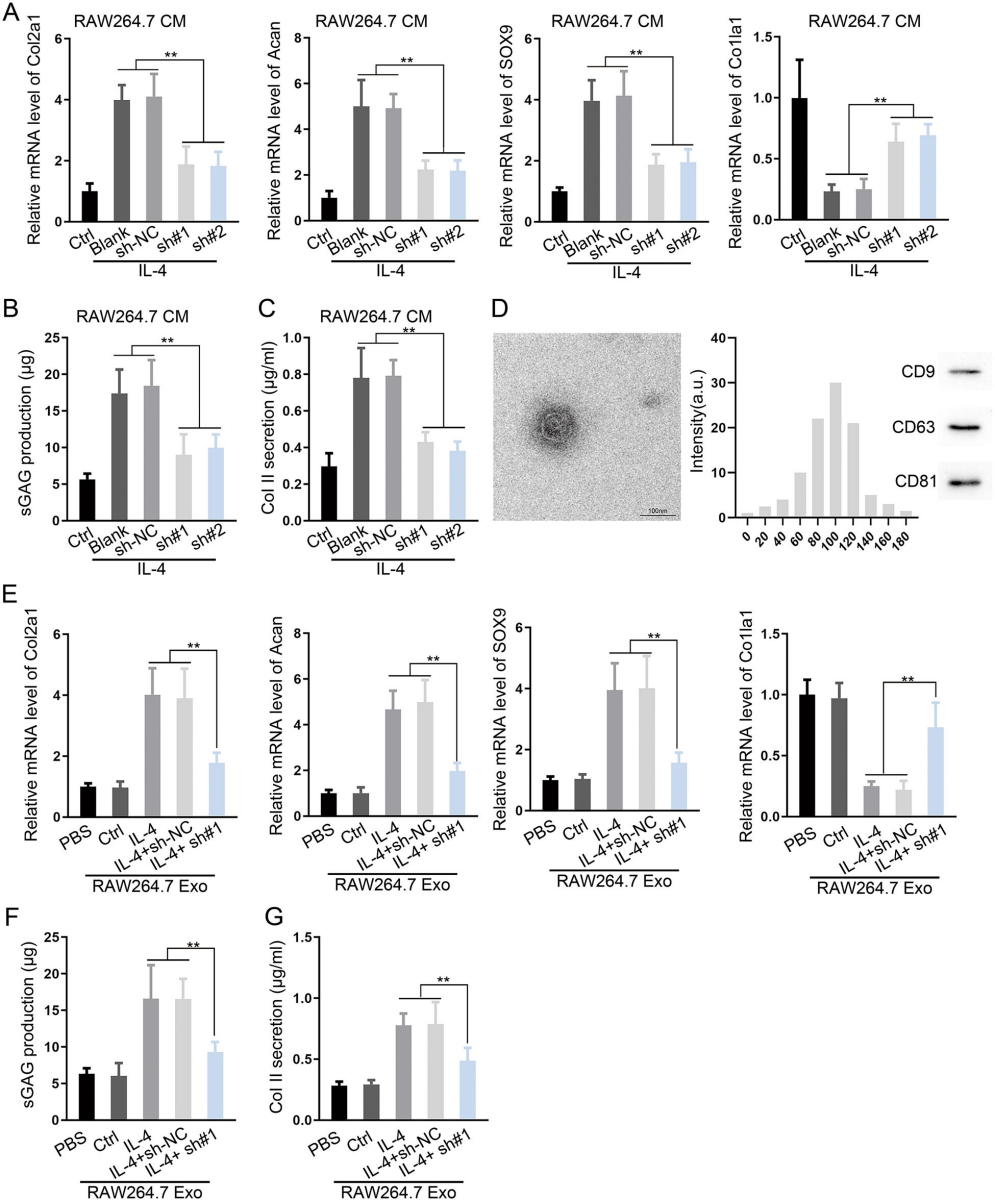
MM2P silence impaired the cartilage repair induced by M2 macrophage-derived exosomes. Chondrocytes were cultured in conditioned medium (CM) of IL-4/IL-13-induced M2 macrophages with indicated transfections. **a** RT-qPCR data of chondrogenic-specific genes Col2a1, Acan, and SOX9, as well as dedifferentiation-related gene Col1a1 of each group. **b**, **c** The production of sGAG and Collagen II in chondrocytes. **d** TEM and nanoparticle tracking analysis (NTA) analysis of exosomes from IL-4/IL-13-induced M2 macrophages. The exosome markers CD9, CD63, and CD81 were detected by western blot. **e** Levels of Col2a1, Acan, SOX9, and Col1a1 in chondrocytes after the incubation with exosomes from IL-13/IL-4-induced M2 macrophages with MM2P silence. **f**, **g** The production of sGAG and Collagen II in each group. **P < 0.01. The error bar expressed as mean ± SD of three independent experiments in triplicates.

RAW264.7 cells induced by IL-13/IL-4 (M2 macrophages) were transfected with sh-MM2P#1/2. M2 macrophages culture medium (CM) was collected to culture with chondrocytes. Chondrogenic-specific genes were downregulated and differentiation-related gene *Col1a1* was upregulated (Fig. 4a and Supplementary Figs. S4A and S5A). The production of sGAG and Col II were reduced (Fig. 4b, c and Supplementary Fig. S4B, C). Similar results were found in BMDMs (Supplementary Figs. S6A–C and S7A–D). By adding the LPS-treated BMDMs CM, chondrogenic-specific genes were downregulated and the differentiation-related gene *Col1a1* was upregulated in chondrocytes (Supplementary Figs. S6D–F and S7E, F).

Next, to confirm the efficacy of exosomes, exosome-free CM from M2 macrophage was added to chondrocytes, the expression of chondrogenic-specific markers were decreased significantly compared with exosomal CM (Supplementary Fig. S8A–C). Exosome extracted from M2 macrophage was added into the medium of chondrocytes. Chondrogenic-specific markers were increased markedly (Figs. 4f, g and 6e and Supplementary Figs. S5B and S9A–C). We further studied the role of exosome in the animal model. Exosomes injection reversed aberrant subchondral bone formation and sclerosis, whereas MM2P-silenced exosomes revealed no cartilage repair evidence (Supplementary Fig. S3).

### MM2P facilitated transactivation of SOX9 in M2 macrophage through STAT3

**Fig. 5:**
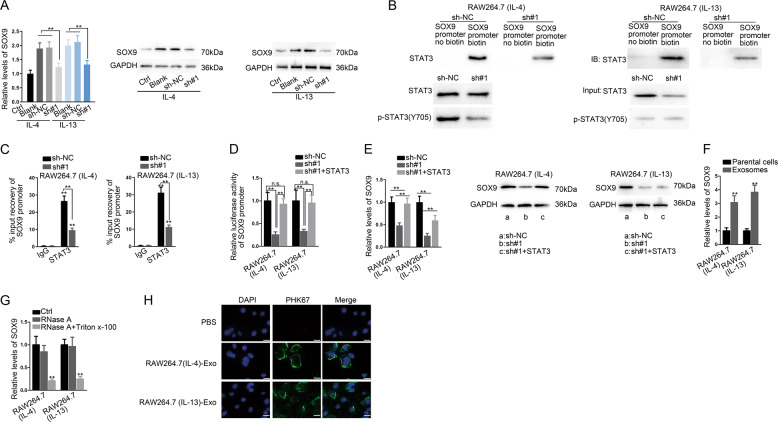
MM2P induced STAT3-regulated transactivation of SOX9 in M2 macrophages and M2 macrophages transmitted SOX9 to chondrocytes. **a** The expression of SOX9 mRNA and protein in RAW264.7 cells under IL-13/IL-4 stimulation after silencing MM2P. **b**, **c** ChIP analysis of SOX9 promoter in IL-13/IL-4 induced M2 macrophages. **d** Luciferase activity of SOX9 promoter in IL-13/IL-4-induced M2 macrophages transfected with sh-NC, sh-MM2P, and sh-MM2P^+^ pcDNA3.1-STAT3. **e** SOX9 expression was detected by RT-qPCR and western blot in each group. **f** Level of SOX9 mRNA in M2 macrophage-derived exosomes and in the parental M2 macrophages. **g** The extracellular expression of SOX9 in the medium of IL-13/IL-4-induced M2 macrophages under the treatment of RNase A and Triton-X 100. **h** The exosomes isolated from IL-13/IL-4-induced M2 macrophages were marked by PKH67. The fluorescence intensity of PKH67 in chondrocytes was detected after culturing with the exosomes. Scale bar = 20 μm. **P < 0.01. The error bar expressed as mean ± SD of three independent experiments in triplicates.

SOX9 expression was upregulated in M2 macrophages, and MM2P silence inhibited it (Fig. 5a and Supplementary Fig. S10A). Knocking down MM2P reduced the enrichment of STAT3 in the SOX9 promoter biotin group (Fig. 5b). MM2P silence decreased SOX9 abundance in STAT3 immunoprecipitates (Fig. 5c). Overexpression of STAT3 increased total STAT3 and p-STAT3 (Y705) except for the p-STAT3/STAT3 ratio (Fig. S11A). Luciferase activity of the SOX9 promoter was inhibited by depleting MM2P, reversed by the overexpression of STAT3 (Fig. 5d). STAT3 partially restored the decrease of SOX9 mRNA expression induced by MM2P depletion (Fig. 5e).

We confirmed that the level of SOX9 was higher in exosomes (Fig. 5f). Compared with RNase A treatment, SOX9 expression decreased under the treatment of Triton-X 100 (Fig. 5g). Fluorescence intensity was observed in chondrocytes incubated with exosomes marked by PKH67 (Fig. 5h). We extracted exosomes after transfecting Cy3-labeled SOX9 mRNA and GFP-SOX9 fusion protein into M2 macrophages and co-cultured with chondrocytes. Detecting Cy3-SOX9 and GFP-SOX9 in chondrocytes (Supplementary Fig. S11B). In addition, SOX9 was knocked down in M2 macrophages (Supplementary Figs. S11C and S12A), resulting in the decrease of chondrogenic-specific genes and protein compared with SOX9 normal expression group (Supplementary Figs. S11D–F and S12B, C).

### MM2P prevented the SHP2-mediated dephosphorylation of STAT3

**Fig. 6:**
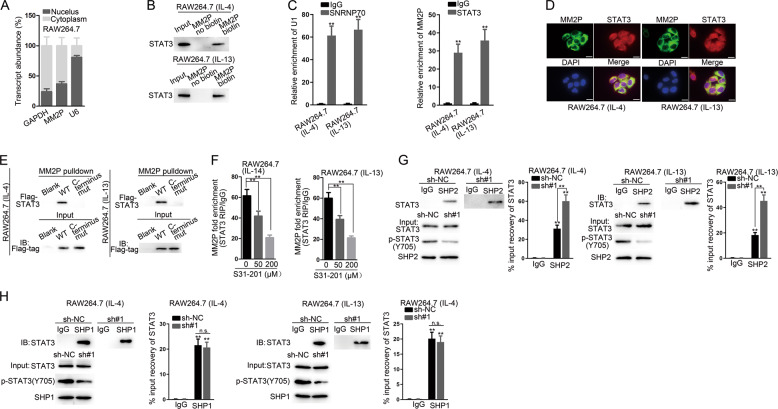
MM2P prevented the SHP2-regulated dephosphorylation of STAT3. **a** Subcellular fractionation assay for the MM2P location in RAW264.7 cells. **b** Pulldown assay was performed to verify the enrichment of STAT3 protein in the products pulled down by the MM2P biotin probe compared with the non-biotin probe. **c** RIP analysis confirmed the interaction between MM2P and STAT3. **d** FISH stain of MM2P and IF stain of STAT3 in M2 macrophages. Scale bar = 20 μm. **e** MM2P**-**pulldown assay for STAT3 enrichment after transfecting Flag-tagged STAT3 into IL-13/IL-4-induced M2 macrophages. **f** RIP analysis was used to detect the interaction between STAT3 and MM2P after adding S31–201 with indicated dose, the inhibitor of STAT3. **g** Co-IP assay was carried out in IL-13/IL-4-induced M2 macrophages to identify the binding of SHP2 or SHP1 with STAT3 after silencing MM2P. **P < 0.01. The error bar expressed as mean ± SD of three independent experiments in triplicates.

MM2P was mainly located in the cytoplasm (Fig. 6a). Pulldown and RIP analysis verified the binding of STAT3 and MM2P (Fig. 6b, c). MM2P and STAT3 co-localized in the cytoplasm (Fig. 6d). Flag-tagged STAT3 was transfected into M2 macrophages and pulldown analysis did not detect the mutated C terminus STAT3 (Fig. 6e), demonstrating MM2P interacted with STAT3 at the C terminus region. S31–201, the inhibitor of STAT3, reduced the binding between MM2P and STAT3 protein in a dose-dependent manner (Fig. 6f). MM2P knockdown increased the binding between SHP2 instead of SHP1 with STAT3 (Fig. 6g, h). Besides, overexpression of MM2P reduced the enrichment of STAT3 in the immunoprecipitate of SHP2, with total STAT3 and SHP2 unchanged (Supplementary Fig. S13A). Overexpression of MM2P attenuated the colocalization of SHP2 and STAT3 in the cytoplasm, whereas knockdown of MM2P augmented it (Supplementary Fig. S13B). Hence, MM2P prevented the SHP2-regulated dephosphorylation of STAT3.

### MM2P interacted with FUS to stabilize SOX9 mRNA

**Fig. 7:**
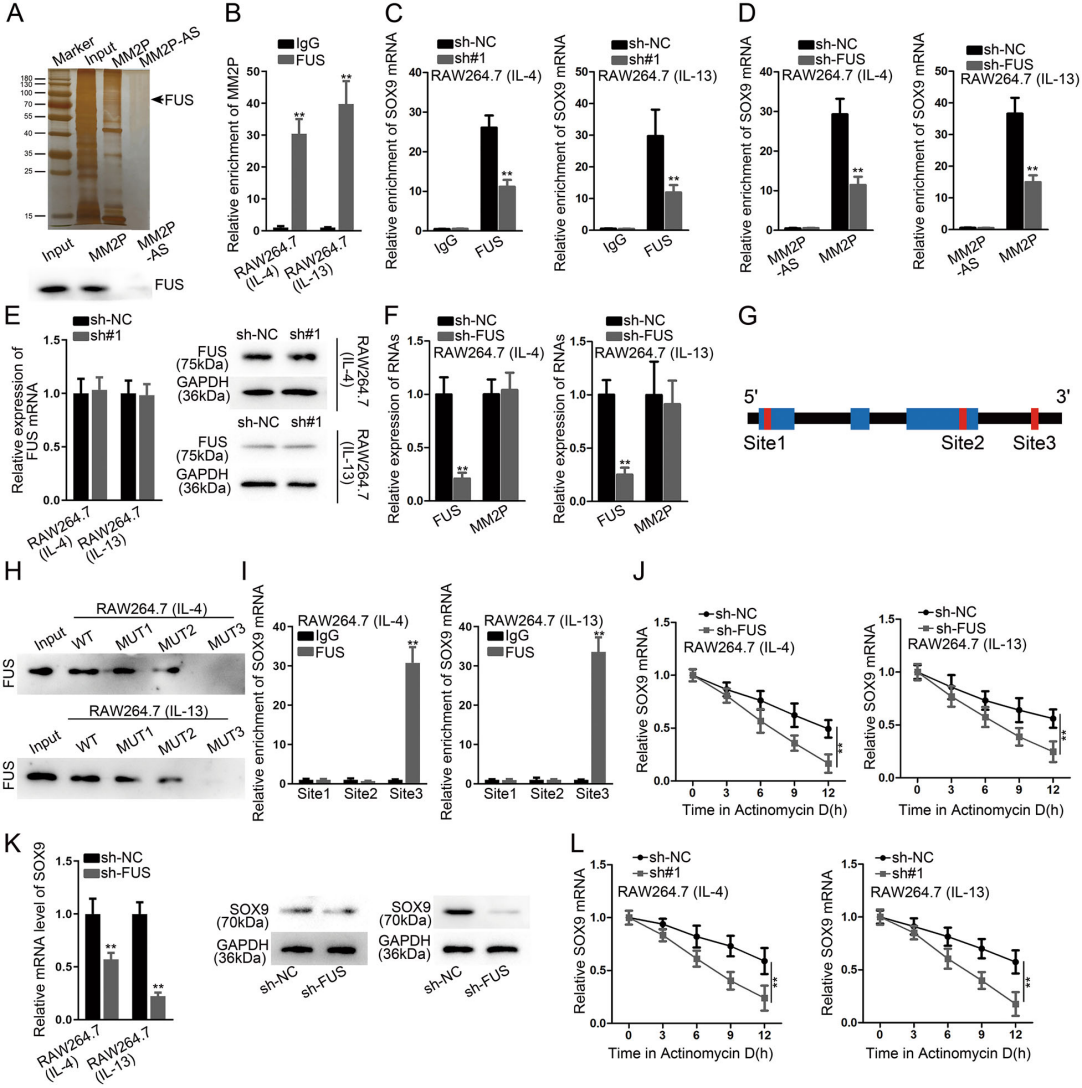
MM2P interacted with FUS to stabilize SOX9 mRNA. **a** Pulldown assay followed by mass spectrometry identified the interaction between FUS and MM2P, which was confirmed by western blot**. b**, **c** RIP assay for the abundance of MM2P in FUS precipitates, and the abundance of SOX9 mRNA in the precipitates of FUS. **d** Pulldown assay for the abundance of SOX9 in the pulldown of the MM2P biotin group after silencing FUS. **e** RT-qPCR and western blot analysis of FUS mRNA and protein levels under MM2P depletion. **f** Levels of FUS and MM2P in IL-13/IL-4-induced M2 macrophages after silencing FUS. **g** Three potential FUS binding sites on SOX9 mRNA predicted by CLIP experimental data from Starbase3.0. **h** Pulldown-western blot assay for the enrichment of FUS in the pulldown of SOX9 mRNA. **i** RIP experiments confirmed the interaction between FUS and SOX9 mRNA at site 3 rather site 1 and 2. **j** SOX9 mRNA stability after Actinomycin D treatment, responding to FUS knockdown. **k** SOX9 mRNA and protein expressions under the silence of FUS in IL-13/IL-4-induced M2 macrophages. **l** mRNA stability of SOX9 in IL-13/IL-4-induced M2 macrophages, in response to MM2P knockdown. **P < 0.01. The error bar expressed as mean ± SD of three independent experiments in triplicates.

We identified the protein bands in the pulldown product of MM2P, and found that FUS interacted with MM2P (Fig. 7a and Supplementary Table 1). MM2P enrichment was increased in FUS immunoprecipitates (Fig. 7b). Silencing MM2P reduced SOX9 mRNA enrichment in FUS immunoprecipitates (Fig. 7c). Silencing FUS reduced the abundance of SOX9 mRNA in the MM2P biotin group (Fig. 7d). FUS mRNA and protein levels exhibited no significant change under MM2P depletion (Fig. 7e and Supplementary Fig. S10B). FUS knockdown did not decrease MM2P expression in M2 macrophages (Fig. 7f). CLIP data from Starbase3.0 (http://Starbase.sysu.edu.cn) identified three potential FUS binding sites on SOX9 mRNA (Fig. 7g). FUS was detectable in the pulldown of SOX9 mRNA with mutation of site 1 and site 2 rather than site 3 (Fig. 7h). FUS interacted with SOX9 mRNA at only site 3 (Fig. 7i). FUS knockdown facilitated SOX9 mRNA degradation with Actinomycin D (Fig. 7j). SOX9 mRNA and protein expression was reduced under the silence of FUS (Fig. 7k and Supplementary Fig. S10C). MM2P knockdown attenuated the mRNA stability of SOX9 (Fig. 7l). In a word, MM2P interacted with FUS to stabilize SOX9 mRNA.

### MM2P promote chondrocyte-specific protein through exosome-derived SOX9

**Fig. 8:**
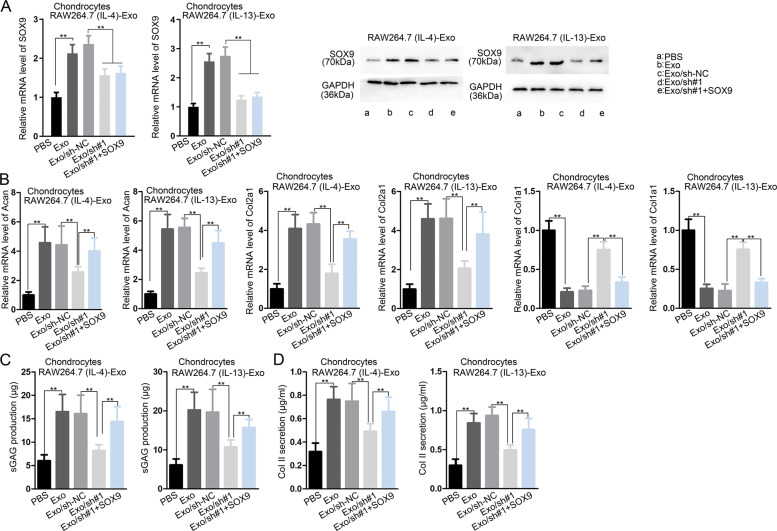
MM2P induced exosomal SOX9 from M2 macrophages to promote cartilage repair. Chondrocytes were cultured with IL-4/IL-13-induced M2 macrophages with indicated transfections. **a** RT-qPCR and western blot experiments for the SOX9 mRNA and protein levels in chondrocytes of each group. **b** Levels of Acan, Col2a1, and Col1a1 in chondrocytes of each group. **c**, **d** The production of sGAG and Collagen II in chondrocytes of each group. **P < 0.01. The error bar expressed as mean ± SD of three independent experiments in triplicates.

M2 macrophages were transfected with sh-NC, sh-MM2P#1, or MM2P#1^+^ pcDNA3.1/SOX9. Exosomes were extracted and co-cultured with chondrocytes. SOX9 mRNA and protein expression were increased in the Exo group compared with the PBS group and decreased in Exo/sh#1 group compared with Exo and Exo/sh-NC groups (Fig. 8a and Supplementary Fig. S10D). Cartilage-specific genes and proteins increased in the Exo group compared with the PBS group. And specific genes and proteins decreased in Exo/sh#1 group compared with Exo/sh-NC group, which could be partly reversed in Exo/sh#1^+^ SOX9 group (Fig. 8b–d and Supplementary Fig. S10E). These findings indicated that MM2P increased cartilage-specific genes and proteins to promote cartilage repair.

## Discussion

OA is a serious disease affecting the health and life quality of the modern population. Although cartilage repair is referred to as a promising approach to treat OA, its mechanism remains elusive^3–5^. In recent years, there is increasing attention on lncRNAs in cartilage repair^12,13^. According to prior research, lncRNA MM2P is identified as the M2 polarization regulator in macrophages^14^. Multiple studies have stated that M2 polarization helped create a pro-chondrogenic atmosphere in the local OA microenvironment^9–11^. Therefore, it is reasonable to deduce that MM2P might positively affect cartilage repair via M2 polarization.

Our study confirmed that MM2P was upregulated during M2 polarization and contributed to the development of the M2 phenotype, which was concordant to the prior study^14^. It has also been proved that MM2P induced p-STAT6, rather than p-STAT1 during M2 polarization^14^. Besides STAT6 and STAT1, many studies also suggested that STAT3 was important to M2 polarization^17,18^. Interestingly, our study found that MM2P activated not only STAT6 but also STAT3. Specifically, we confirmed that MM2P induced p-STAT3 (Y705) rather than p-STAT3 (S727) (Fig. 1f and Supplementary Fig. S1F), indicating that MM2P also regulated M2 polarization through STAT3 signaling. Since the regulation of MM2P on STAT6 in M2 polarization has been validated, we focused on its impacts on STAT3 signaling in the current study.

Functionally, we proved that M1 macrophages differentiated from BMDMs inhibited the functions of chondrocytes, in which MM2P could promote the M1-to-M2 polarization in BMDMs (Figs. 2e and 3c–g). Recent studies have revealed that exosomes mediated intracellular communication^28,29^. Chondrocytes could release exosome-like vesicles to affect IL-1β production of macrophages and aggravate OA progression^30^. Our study showed that M2 macrophages exosome-free CM exhibited much weaker effects on cartilage repair in vitro, indicating that the influence of M2 macrophages on cartilage repair depended on the exosomes partially (Fig. 4a–c and Supplementary Fig. S8). We later proved that MM2P mediated the influence of M2 macrophage-released exosomes on chondrocyte functions, which might potentially promote cartilage repair. In vivo data further supported the results (Supplementary Fig. S3).

A previous study showed that STAT3 could combine to the proximal promoter of SOX9 and trigger its transcription in chondrocytes^22^. SOX9 is axiomatically recognized as the master modulator of many chondrogenic genes, including *Acan*, *Col2a1*, and *Col1a1*^23–25^. Hence, we speculated that MM2P could regulate SOX9 via STAT3, and SOX9 could be transferred by exosomes from M2 macrophages to chondrocytes in cartilage repair. We showed that MM2P demonstrated increased binding and transactivation of STAT3 on SOX9 by inducing p-STAT3 (Y705) (Fig. 5a–e). We also explored the MM2P regulation to p-STAT3 (Y705). A previous study proved that MM2P regulated p-STAT6 by the tyrosine phosphatase inhibitor Na_3_VO_4_^34^, and suggested that cytoplasmic MM2P might also regulate STAT6 phosphorylation through regulating the SHP1 or SHP2 interaction with STAT6. Another study demonstrated that lnc-DC promoted STAT3 phosphorylation through prohibiting SHP1 via binding to the C terminus to STAT3^35^. SHP1 and SHP2, belong to PTPs, are responsible for the dephosphorylation and inactivation of STAT3^21^. We verified that MM2P could interact with STAT3 at the C terminus region that contains Y705 residue^36^ (Fig. 6e). Interestingly, we showed that MM2P inhibited the interaction of STAT3 with SHP2 rather than SHP1 (Fig. 6g, h and Supplementary Fig. S13), suggesting that MM2P regulated SHP2-mediated dephosphorylation of STAT3.

As previously reported, lncRNAs cooperated with RNA-binding proteins to affect target genes^37,38^. Our study first uncovered through pulldown that FUS was a partner with MM2P (Fig. 7a). FUS is a multifunctional protein regulating transcription, RNA splicing, and RNA stability depending on its cellular localization^39,40^. Several studies also delineated that lncRNA coupled with FUS to regulate downstream genes^41,42^. Here, we revealed that MM2P interacted with FUS to stabilize SOX9 mRNA in M2 macrophages. We also showed that MM2P induced M2 macrophage-derived SOX9 could promote chondrocytes (Fig. 8), indicating that MM2P might influence cartilage repair through SOX9-induced chondrocytes.

## Conclusions

We demonstrated that lncRNA MM2P significantly enhanced the functions of chondrocytes by inducing M2 macrophage polarization, and promoting the transmission of M2-derived exosomal SOX9 into chondrocytes (Supplementary Fig. S14). Our data clearly illustrated the therapeutic potential of targeting MM2P as a novel promising approach to treat OA through promoting cartilage repair. This study supported a novel therapeutic strategy for OA.

## Supplementary information

Supplementary figure and table legends

Figure S1

Figure S2

Figure S3

Figure S4

Figure S5

Figure S6

Figure S7

Figure S8

Figure S9

Figure S10

Figure S11

Figure S12

Figure S13

Figure S14

Supplementary table 1
