# Supplementary material for: LncRNA MM2P-induced, exosome-mediated transfer of Sox9 from monocyte-derived cells modulates primary chondrocytes
Source: Cell Death Dis. 2020 Sep 16;11(9):763. doi: 10.1038/s41419-020-02945-5 (PMC7494881; doi:10.1038/s41419-020-02945-5)
Supplement: Supplementary file 1 — Supplementary figure and table legends [file 41419_2020_2945_MOESM1_ESM.docx]

**Supplementary figure and table legends**

**Figure S1. MM2P facilitated M2 polarization. A.** Flow cytometry analysis for the ratio of F4/80+CD206+ in RAW264.7 cells after treatment with IL-13. **B.** Level of MM2P following the induction of M2 polarization at indicated time. **C.** The effect of sh-MM2P#1/2 transfection on the expression of MM2P in IL-13-induced M2 macrophages was measured by RT-qPCR. **D.** Pictures of flow cytometry of F4/80+CD206+ cells and quantification of F4/80+CD206+ cell ratio under MM2P depletion in IL-13 -induced cells. **E.** RT-qPCR data for the levels of M2-related genes (Fizz-1, Arg1, YM1, MRC1, PPAR-γ) under the treatment of IL-13 with MM2P depletion in RAW264.7 cells. **F.** Western blot for the total and phosphorylated STAT1, STAT6 and STAT3 in RAW264.7 cells treated with IL-13. **G.** IF staining for the fluorescence intensity of p-STAT3 under IL-13 stimulation, as well as depletion of MM2P. Scale bar = 20 μm. ^**^P < 0.01. Error bars are said to express as mean ± SD of 3 independent experiments in triplicates.

**Figure S2.** RT-qPCR analysis of Figure 1B, 1C, and 1E using ACTB and 18S as housekeeping genes.

**Figure S3.** HE staining of the knee joints collected from mice injected with PBS, Exo, Exo/sh-NC and Exo/sh-MM2P. Sham group was taken as the negative control. Scale bar = 100 μm. Safranin O staining of the knee joints in different groups. Scale bar = 100 μm.

**Figure S4 Effect of MM2P on chondrogenic genes.** Chondrocytes were cultured in conditioned medium (CM) of IL-13-induced M2 macrophages with indicated transfections. **A.** RT-qPCR data of chondrogenic specific genes Col2a1, Acan, and SOX9, as well as dedifferentiation-related gene Col1a1 of each group. **B-C.** The production of sGAG and Collagen II in chondrocytes. ^**^P < 0.01. Error bar expressed as mean ± SD of 3 independent experiments in triplicates.

**Figure S5** RT-qPCR analysis of Figure 2A and 2E using ACTB and 18S as housekeeping genes.

**Figure S6. M2 macrophages induced cartilage repair in chondrocytes.** The CM of BMDMs of indicated groups were applied to culture chondrocytes. **A, D.** RT-qPCR analysis of Col2a1, Acan, SOX9, and Col1a1 in chondrocytes cultured with BMDMs of indicated groups. **B, E.** Quantification of sGAG production in chondrocytes cultured with BMDMs of indicated groups. **C, F.** Quantification of Col II secretion of chondrocytes cultured with BMDMs of indicated groups. ^**^P < 0.01. Error bar expressed as mean±SD of 3 independent experiments in triplicates.

**Figure S7.** RT-qPCR analysis of Figure S2A and S2D using ACTB and 18S as housekeeping genes.

**Figure S8. Exosomes derived from M2 macrophages were required for the effect of M2 macrophage on cartilage repair.** Chondrocytes were cultured in the RAW264.7/IL-4-CM or exosome-free RAW264.7/IL-4-CM. **A.** RT-qPCR of Col2a1, SOX9, Acan, and Col1a1 in chondrocytes of indicated groups, with GAPDH, ACTB, and 18S as housekeeping genes respectively. **B-C.** Quantification of Col II secretion of chondrocytes of indicated groups. ^**^P < 0.01. Error bar expressed as mean ± SD of 3 independent experiments in triplicates.

**Figure S9. Effect of M2 macrophage-derived exosomal MM2P on chondrogenic genes. A.** Levels of Col2a1, Acan, SOX9 and Col1a1 in chondrocytes after the incubation with exosomes from IL-13-induced M2 macrophages with MM2P silence. **B-C.** The production of sGAG and Collagen II in each groups. ^**^P < 0.01. Error bar expressed as mean ± SD3 independent experiments in triplicates.

**Figure S10.** RT-qPCR analysis of Figure 3A, 5E, 5K, 6B, 6A, and 6B using ACTB and 18S as housekeeping genes.

**Figure S11.** **A.** Western blots of p-STAT3 and STAT3 and quantification of p-STAT3/STAT3 in RAW264.7 transfected with pcDNA3.1 or pcDNA3.1/STAT3. **B.** IL-4-treated RAW264.7 cells were transfected with Cy3-labeled SOX9 mRNA or GFP-SOX9 fusion protein. The exosomes derived from IL-4-treated RAW264.7 cells were extracted and added to the medium of chondrocytes. The representative pictures of the fluorescence of Cy3-SOX9 mRNA and GFP-SOX9 were detected in chondrocytes cultured with exosomes or PBS control. Scale bar = 10 μm. **C.** RT-qPCR and western blot analysis of SOX9 in RAW264.7 cells of indicated groups. **D.** Chondrocytes were cultured with PBS control or RAW264.7 (IL-4)-Exo with sh-NC or sh-SOX9#1/2. RT-qPCR analysis of SOX9, Acan, Col2a1, and Col1a1 levels in chondrocytes of each group. **E-F.** Quantification of Col II secretion of chondrocytes of indicated groups. ^**^P < 0.01. Error bar expressed as mean ± SD of 3 independent experiments in triplicates.

**Figure S12.** RT-qPCR analysis of Figure S4C, S4D, and S4E using ACTB and 18S as housekeeping genes.

**Figure S13. A.** Western blot of STAT3 in the IP of SHP2 and STAT3, p-STAT3, and SHP2 in input in RAW264.7 cells transfected with pcDNA3.1 or pcDNA3.1/MM2P. The enrichment of STAT3 in the IP of SHP2 was quantified. **B.** IF staining of the overlap of SHP2 and STAT3 in RAW264.7 cells transfected with pcDNA3.1 or pcDNA3.1/MM2P or in the IL-4-treated RAW264.7 cells transfected with sh-NC or sh-MM2P#1. Scale bar = 10 μm. ^**^P < 0.01. Error bar expressed as mean ± SD of 3 independent experiments in triplicates.

**Figure S14 Graphical abstract.** lncRNA MM2P promoted chondrocyte functions by inducing M2 polarization to promote the transmission of M2 macrophage-derived exosomal SOX9 into chondrocytes. MM2P prevented SHP2-regulated de-phosphorylation of STAT3 to activate STAT3 and interacted with FUS to stabilize STAT3 mRNA in M2 macrophages.

**Table S1 Original data for mass spectrometry**
