# Supplementary figures and images for: LncRNA MM2P-induced, exosome-mediated transfer of Sox9 from monocyte-derived cells modulates primary chondrocytes
Source: Cell Death Dis. 2020 Sep 16;11(9):763. doi: 10.1038/s41419-020-02945-5 (PMC7494881; doi:10.1038/s41419-020-02945-5)

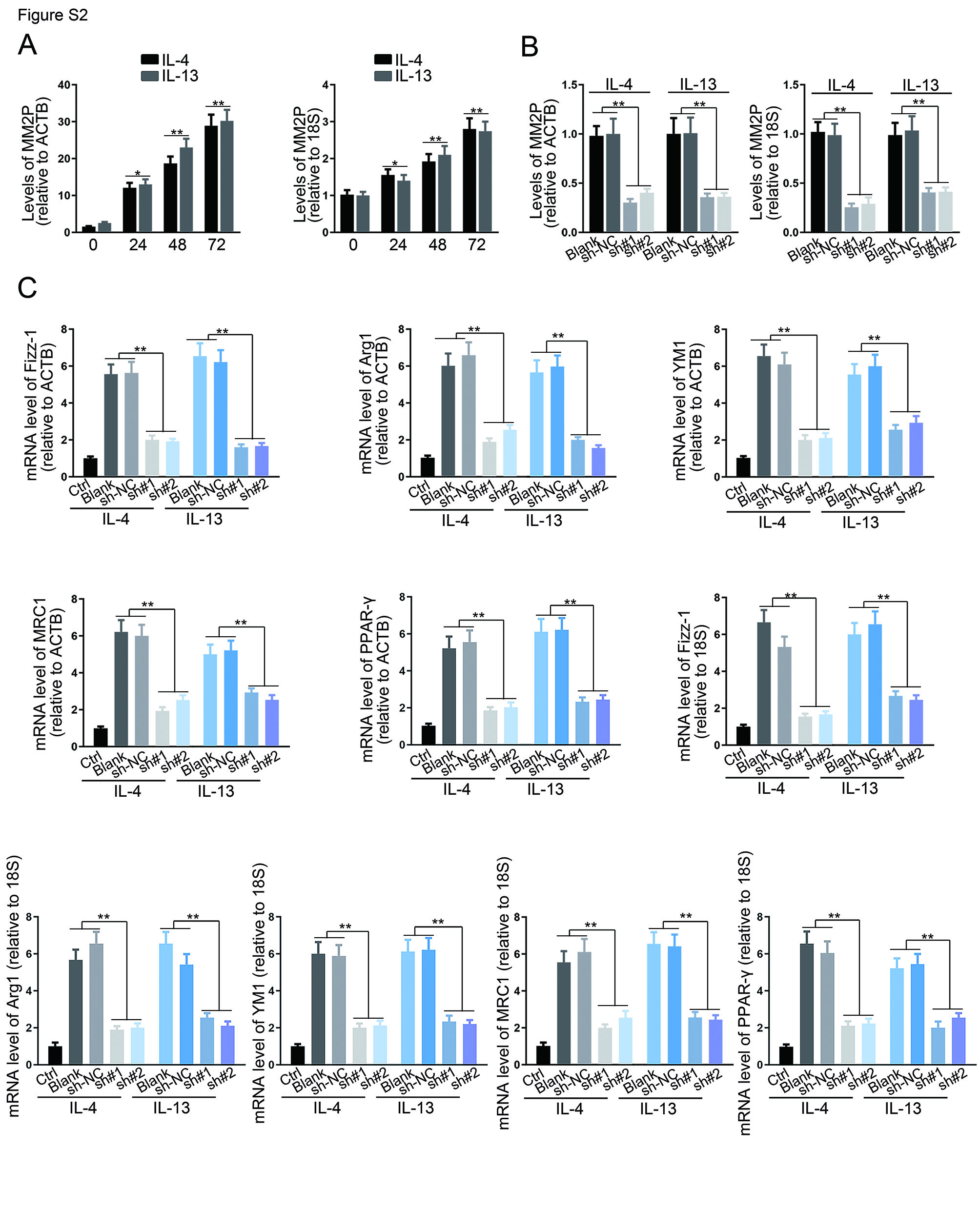

Supplement: Supplementary file 3 — Figure S2 [file 41419_2020_2945_MOESM3_ESM.tif]

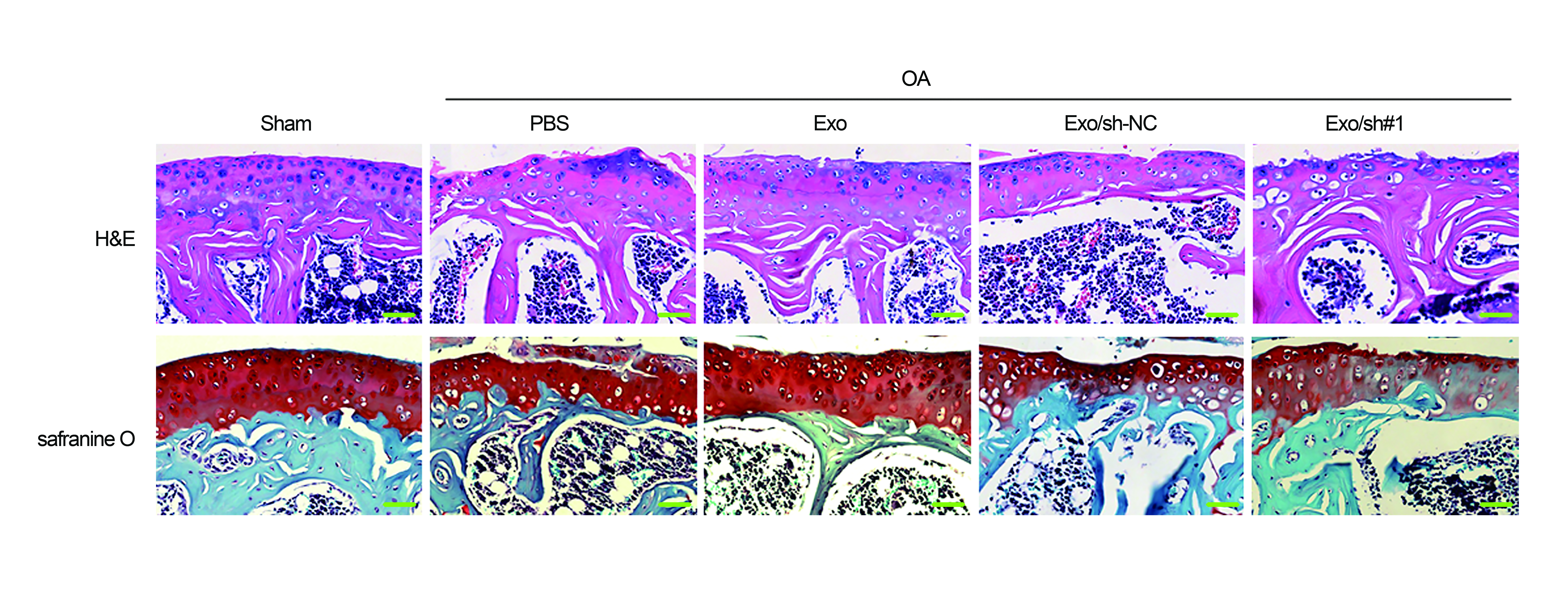

Supplement: Supplementary file 4 — Figure S3 [file 41419_2020_2945_MOESM4_ESM.tif]

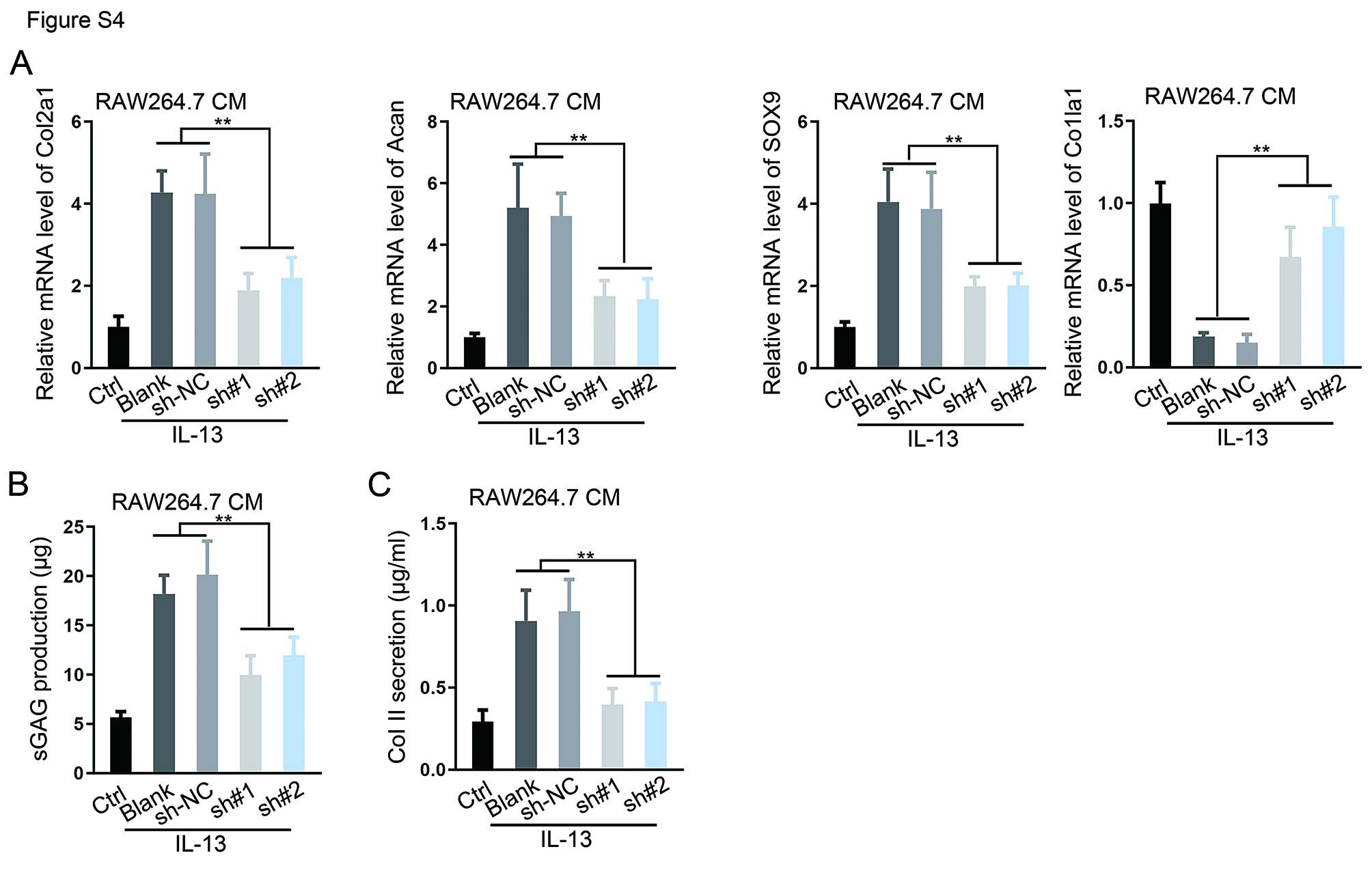

Supplement: Supplementary file 5 — Figure S4 [file 41419_2020_2945_MOESM5_ESM.tif]

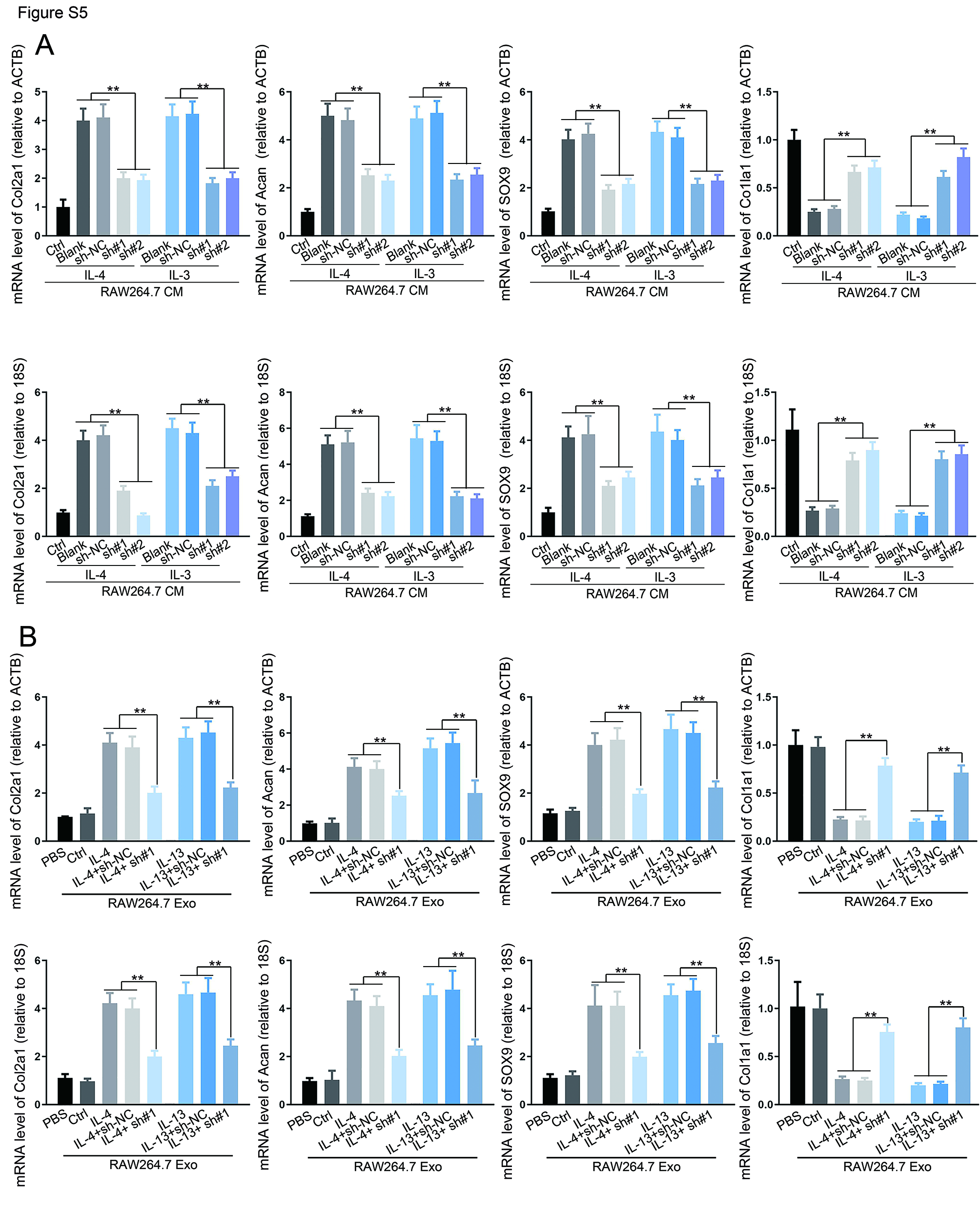

Supplement: Supplementary file 6 — Figure S5 [file 41419_2020_2945_MOESM6_ESM.tif]

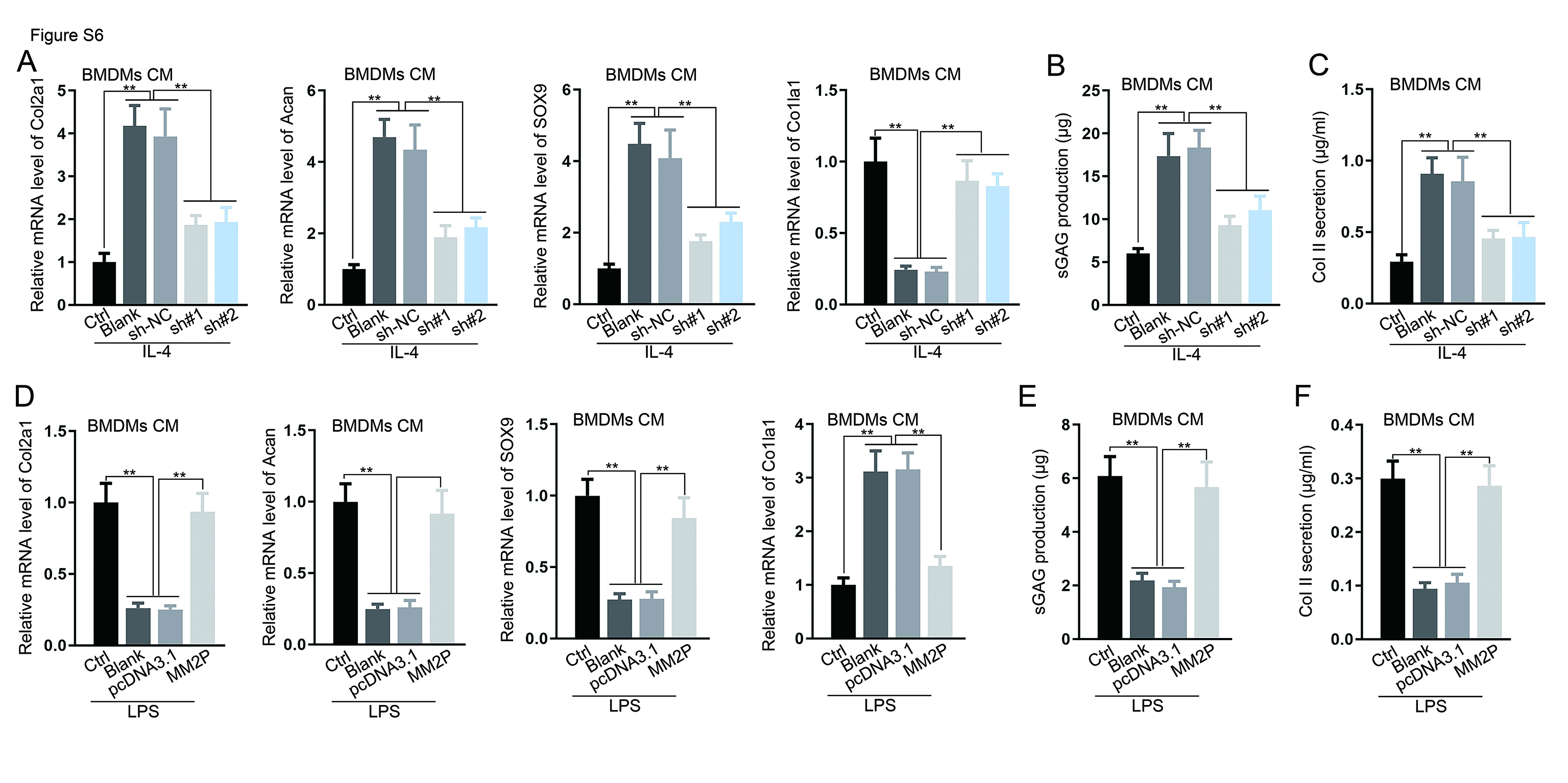

Supplement: Supplementary file 7 — Figure S6 [file 41419_2020_2945_MOESM7_ESM.tif]

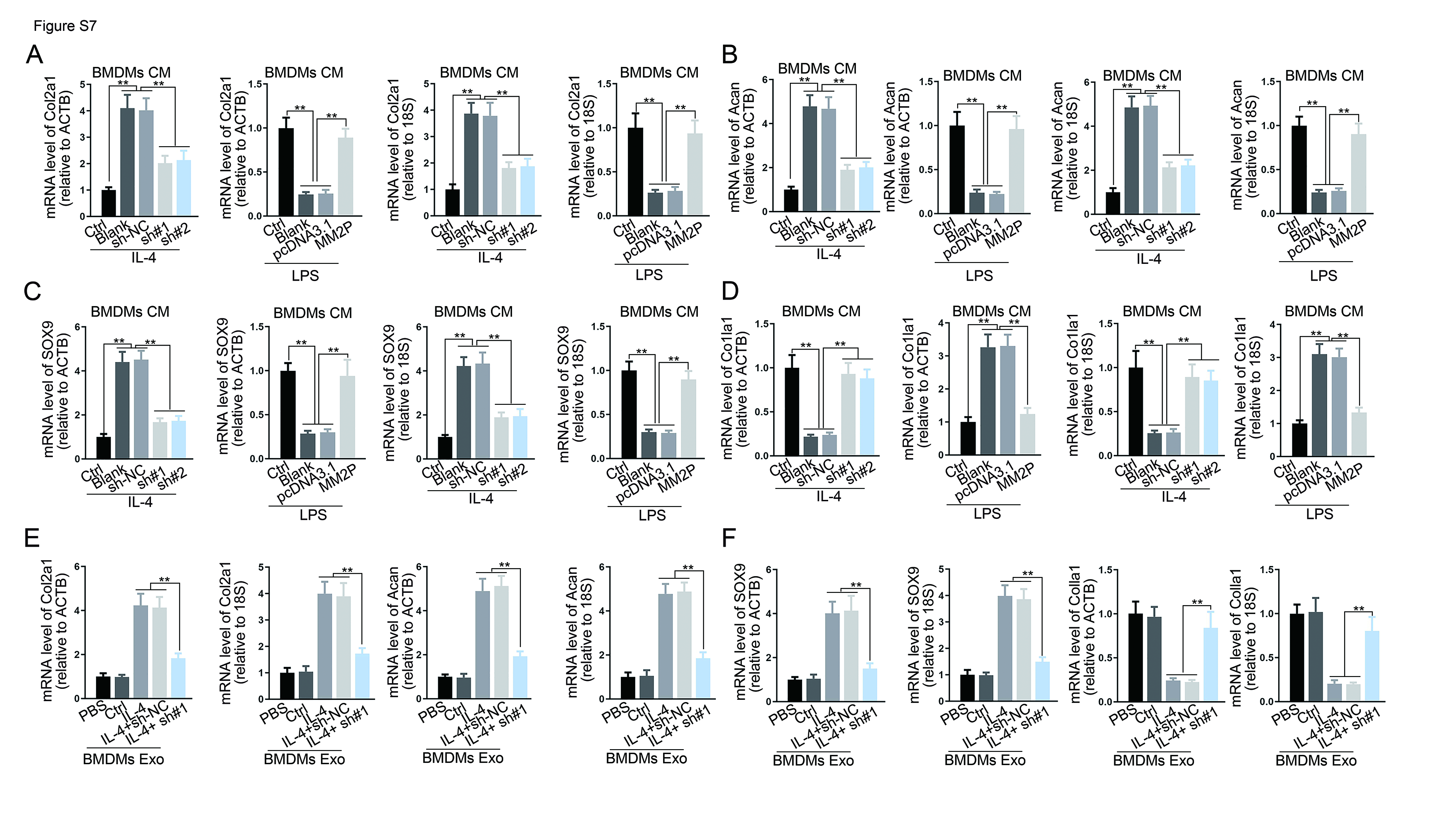

Supplement: Supplementary file 8 — Figure S7 [file 41419_2020_2945_MOESM8_ESM.tif]

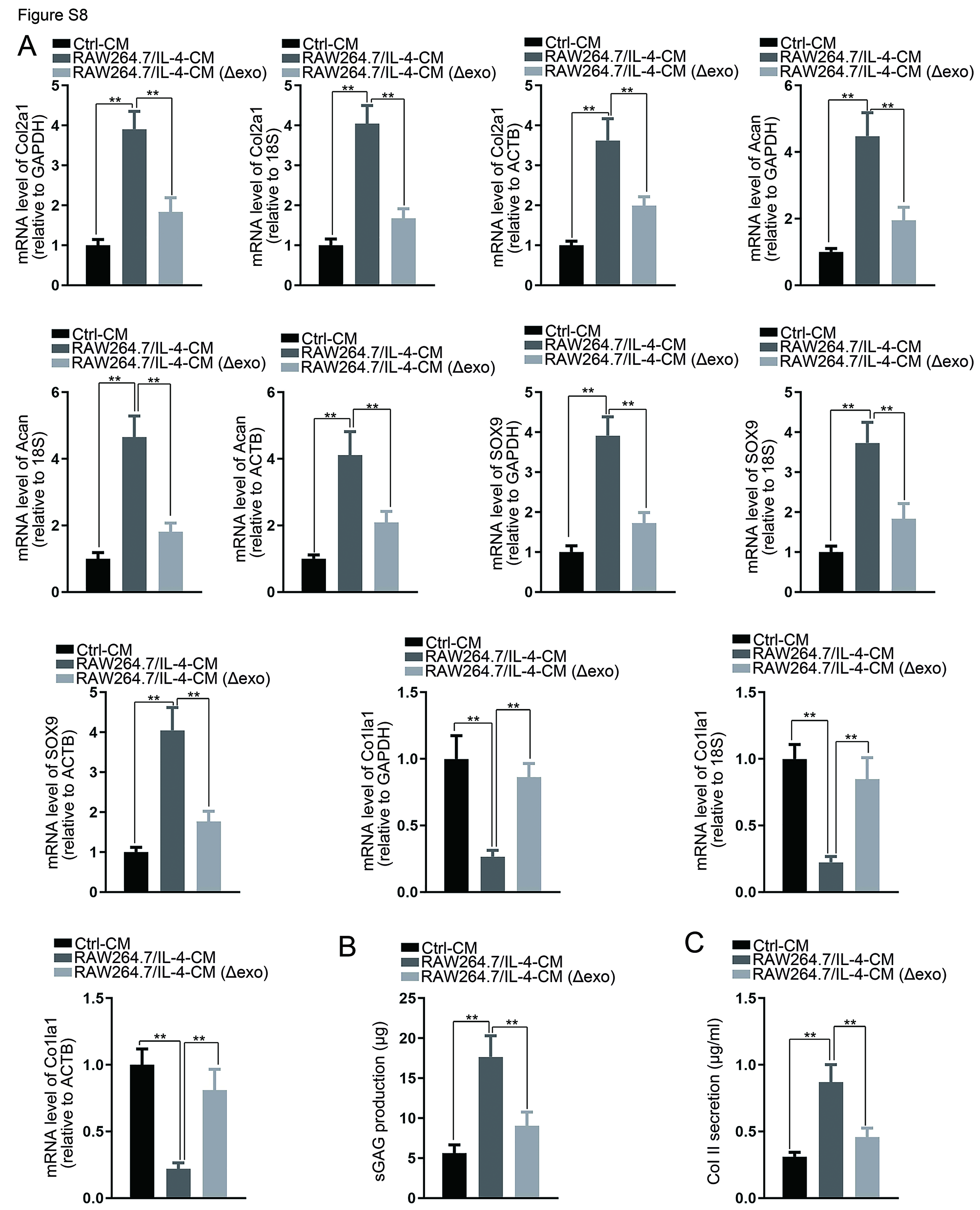

Supplement: Supplementary file 9 — Figure S8 [file 41419_2020_2945_MOESM9_ESM.tif]

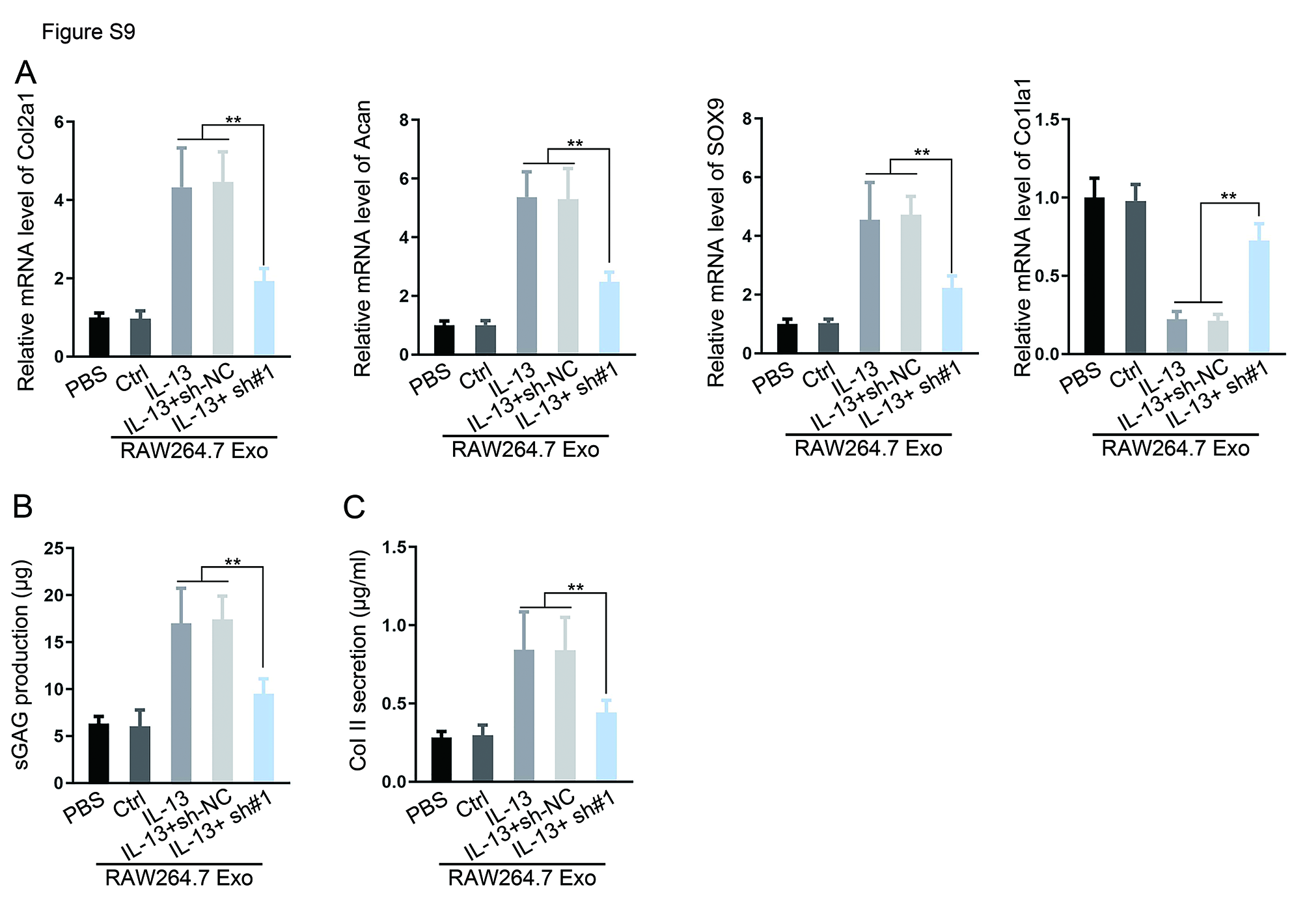

Supplement: Supplementary file 10 — Figure S9 [file 41419_2020_2945_MOESM10_ESM.tif]

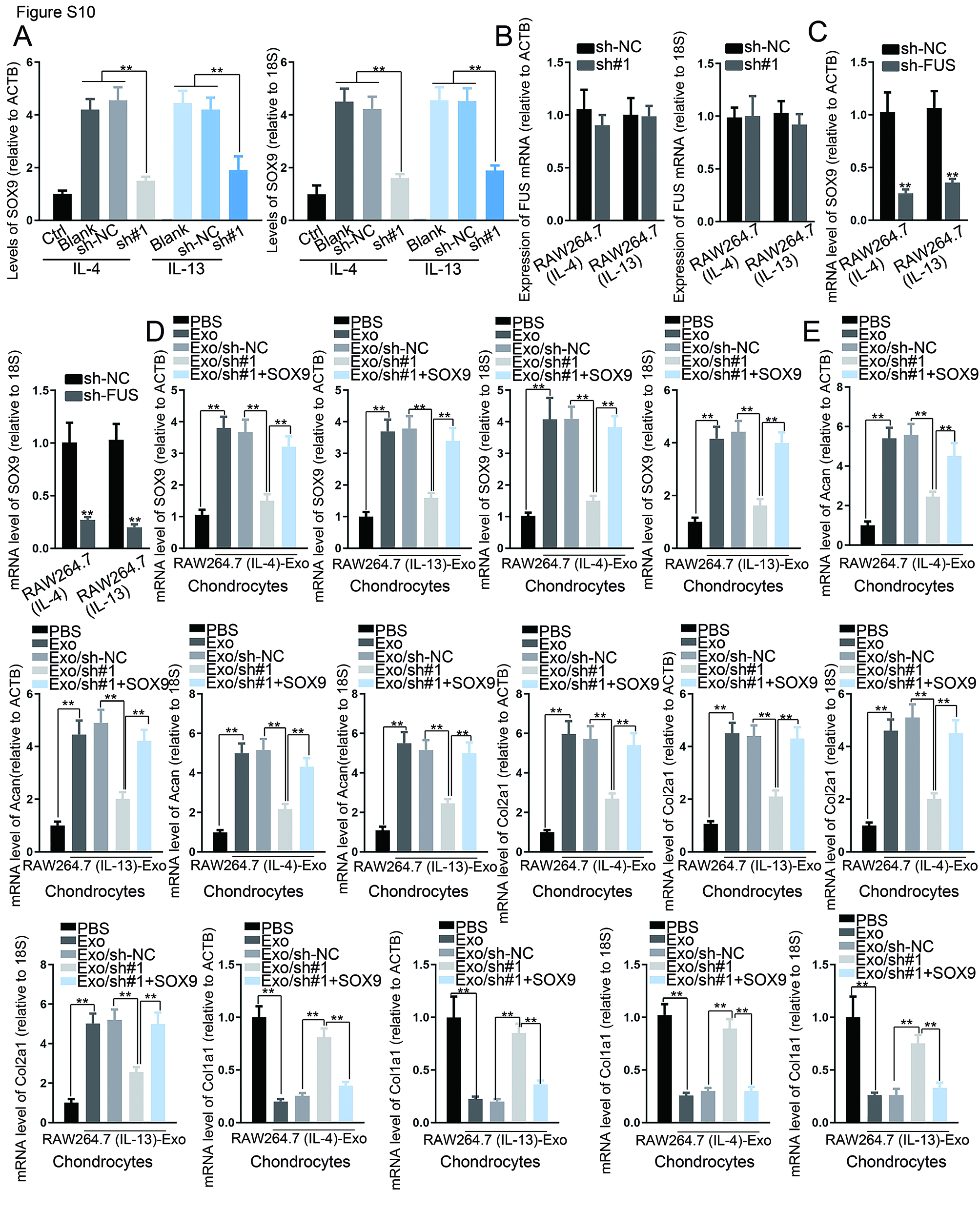

Supplement: Supplementary file 11 — Figure S10 [file 41419_2020_2945_MOESM11_ESM.tif]

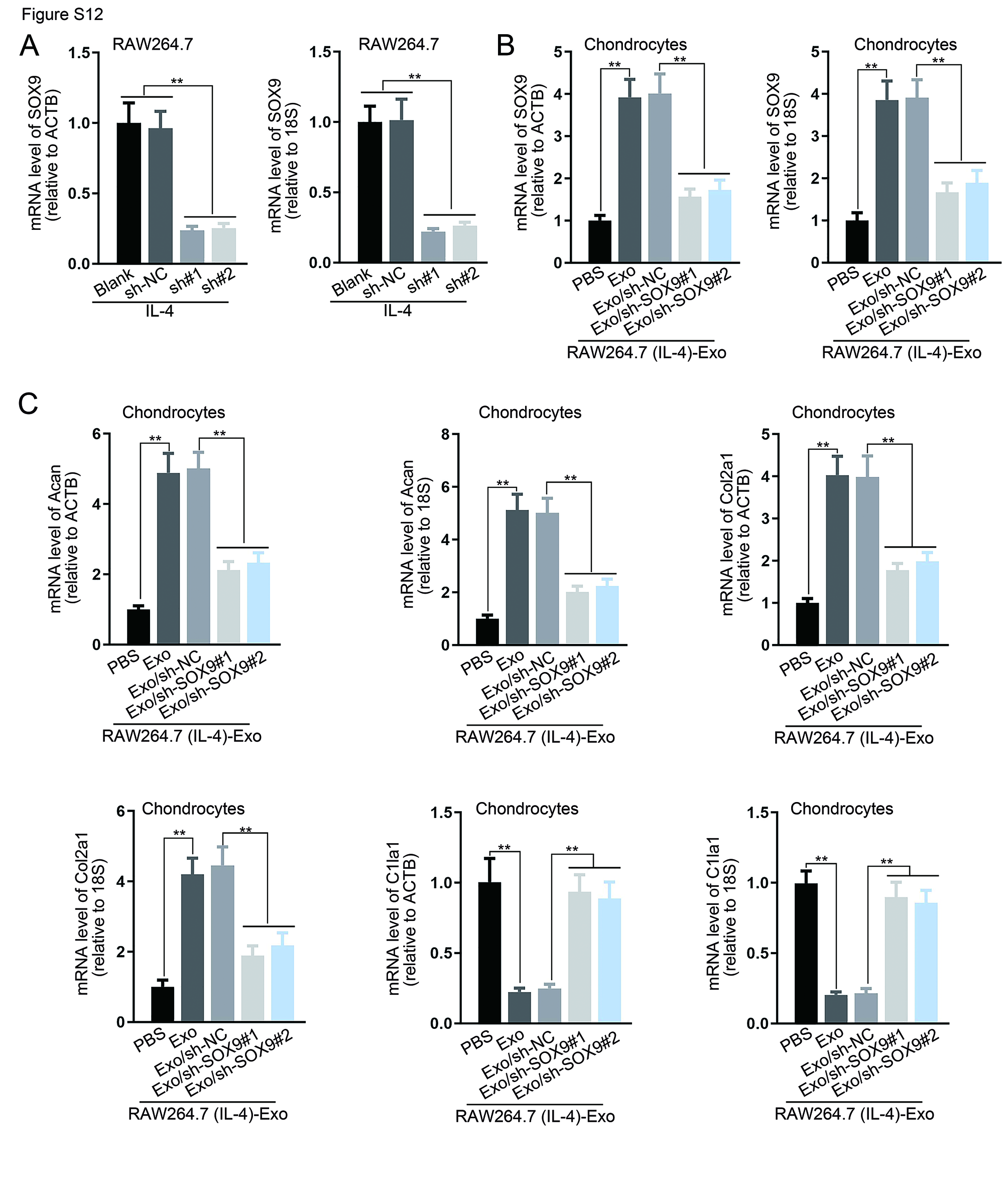

Supplement: Supplementary file 13 — Figure S12 [file 41419_2020_2945_MOESM13_ESM.tif]

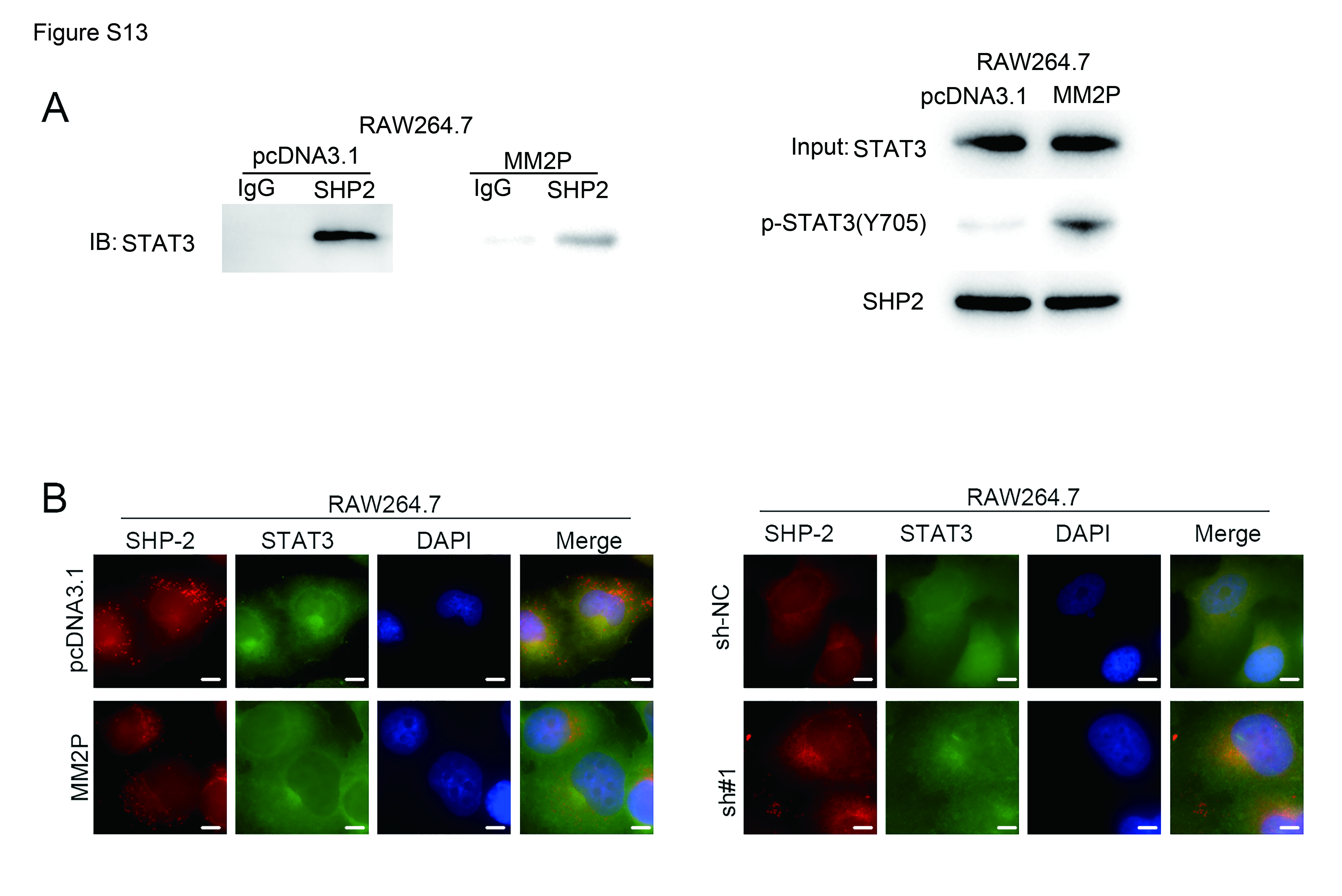

Supplement: Supplementary file 14 — Figure S13 [file 41419_2020_2945_MOESM14_ESM.tif]

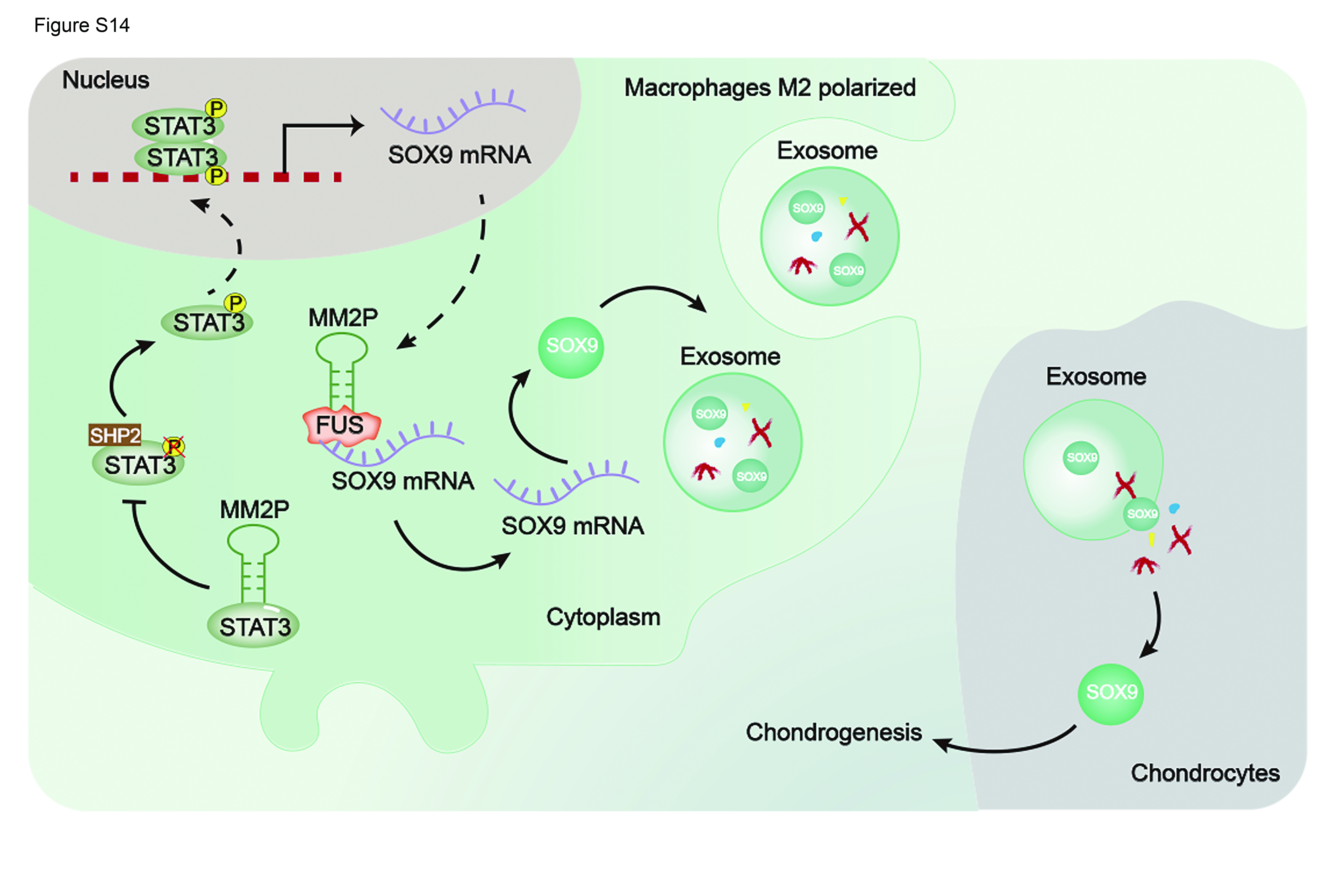

Supplement: Supplementary file 15 — Figure S14 [file 41419_2020_2945_MOESM15_ESM.tif]
